# Can tDCS enhance item-specific effects and generalization after linguistically motivated aphasia therapy for verbs?

**DOI:** 10.3389/fnbeh.2015.00190

**Published:** 2015-07-30

**Authors:** Vânia de Aguiar, Roelien Bastiaanse, Rita Capasso, Marialuisa Gandolfi, Nicola Smania, Giorgio Rossi, Gabriele Miceli

**Affiliations:** ^1^International Doctorate in Experimental Approaches to Language And Brain (IDEALAB, Universities of Trento, Groningen, Potsdam, Newcastle and Macquarie University)Rovereto, Italy; ^2^Center for Mind/Brain Sciences and Center for Neurocognitive Rehabilitation, University of TrentoRovereto, Italy; ^3^Center for Language and Cognition Groningen, University of GroningenGroningen, Netherlands; ^4^S.C.A. AssociatesRome, Italy; ^5^Neuromotor and Cognitive Rehabilitation Research Centre, USO Neurological Rehabilitation, Azienda Ospedaliera Universitaria Integrata (AOUI) of VeronaVerona, Italy; ^6^Department of Neurological and Movement Sciences, University of VeronaVerona, Italy; ^7^Neurology, Santa Maria del Carmine HospitalRovereto, Italy

**Keywords:** aphasia rehabilitation, verb retrieval, argument structure, sentence production, generalization, linguistically motivated therapy, transcranial direct current stimulation (tDCS), neuromodulation

## Abstract

**Background:** Aphasia therapy focusing on abstract properties of language promotes both item-specific effects and generalization to untreated materials. Neuromodulation with transcranial Direct Current Stimulation (tDCS) has been shown to enhance item-specific improvement, but its potential to enhance generalization has not been systematically investigated. Here, we test the efficacy of ACTION (a linguistically motivated protocol) and tDCS in producing item-specific and generalized improvement in aphasia.

**Method:** Nine individuals with post-stroke aphasia participated in this study. Participants were pre-tested with a diagnostic language battery and a cognitive screening. Experimental tasks were administered over multiple baselines. Production of infinitives, of finite verbs and of full sentences were assessed before and after each treatment phase. Nonword repetition was used as a control measure. Each subject was treated in two phases. Ten daily 1-h treatment sessions were provided per phase, in a double-blind, cross-over design. Linguistically-motivated language therapy focusing on verb inflection and sentence construction was provided in both phases. Each session began with 20 min of real or sham tDCS. Stimulation site was determined individually, based on MRI scans.

**Results:** Group data showed improved production of treated and untreated verbs, attesting the efficacy of behavioral treatment, and its potential to yield generalization. Each individual showed significant item-specific improvement. Generalization occurred in the first phase of treatment for all subjects, and in the second phase for two subjects. Stimulation effects at the group level were significant for treated and untreated verbs altogether, but a ceiling effect for Sham cannot be excluded, as scores between real tDCS and Sham differed only before treatment.

**Conclusion:** Our data demonstrate the efficacy of ACTION and suggest that tDCS may enhance both item-specific effects and generalization.

## Introduction

Aphasia is an acquired language disorder that occurs following brain damage, frequently caused by stroke, traumatic brain injury or brain tumors. Though different rehabilitation strategies have been used in aphasia, they all share the general aim of improving communication. Currently available evidence indicates that aphasia therapy is effective (Brady et al., 2012). Nevertheless, 43% of the individuals with aphasia who suffer from language disorders due to a first-ever stroke are still aphasic 18 months post-onset (Laska et al., 2001). While most research on aphasia therapy focuses on the recovery of nouns, there is an increasing interest in the rehabilitation of verb and sentence production (Webster and Whitworth, 2012). Research addressing how to optimize verb and sentence rehabilitation programs to produce larger item-specific effects and generalization is needed. A recent addition to treatment tools for aphasia rehabilitation is tDCS—a neuromodulation technique introduced to increase treatment efficacy, in combination with Speech-Language Therapy. tDCS may enhance item-specific improvement, and it seems to be effective across a variety of tasks (de Aguiar et al., 2015).

The current study has three main goals. First, to test the efficacy of the Italian version of ACTION, a treatment protocol shown to result in generalization (Bastiaanse et al., 2006; Links et al., 2010). We focus specifically on verb retrieval and inflection in sentence production, and assess the effects of treatment on both treated and untreated verbs. Second, to test the potential of tDCS in enhancing both item-specific improvement and generalization, when paired with ACTION. Third, to discuss individual outcomes in relation to group results, in order to better understand the effects of a treatment combining tDCS and ACTION. In this introduction, we describe the cognitive processes involved in verb and sentence production, and we provide an overview of studies focusing on the treatment of verb and sentence production, and of studies using tDCS.

### Verb and sentence production

A unique feature of lexical representations of verbs is that, contrary to most nouns, they contain information about argument structure that is necessary for sentence production (Saffran et al., 1980). This means that deficits in verb processing may contribute to deficits in sentence processing (e.g., patient HW, Caramazza and Hillis, 1991), though sentence-level deficits may also arise from other types of impairment. The speech-error model (Garrett, 1980) defines 3 processing levels involved in producing sentences. The *message level* entails the speaker's communicative goal and is a nonlinguistic representation of the idea to be conveyed by the speaker. This idea becomes semantically and thematically specified at the *functional level*. Here, semantic word representations are retrieved, the predicate-argument structure of the main verb specifies the number of arguments and the thematic roles required by the verb, and thematic roles are assigned to semantic representations (Schwartz, 2013). Inflectional affixes are included in this syntactic frame (Garrett, 1980). Finally, sentence constituents are ordered, and phonologically specified representations (lexemes) are retrieved from the phonological output lexicon, at the *positional level*. With languages having a limited amount of possible predicate argument structures, there is evidence that different verbs share combinatorial nodes (i.e., the stored information about the syntactic structures in which they occur; Pickering and Branigan, 1998) and that recent exposure to a sentence structure may facilitate the production of the same structure with a different verb (a phenomenon known as structural priming; Bock, 1986).

Even though these levels are conceived of as distinct processing stages, interactions between them are also assumed. For instance, after a syntactic frame is specified, some lexemes are more likely to be activated, due to their relation to appropriate semantic features (Bock, 1986). In addition, evidence for a relation between verb inflection and retrieval was reported by Bastiaanse (2011): individuals with fluent aphasia performed below norm in verb retrieval when producing finite verbs, but they were unimpaired when producing infinitives. Hence, syntax can influence lexical verb retrieval, due to both introduced lexical selection biases, and increased task complexity.

The neural correlates of these processes have been investigated in neuro-imaging research. Verb naming has been associated with activity in dorsolateral frontal and lateral temporal cortex (Perani et al., 1999), left frontal operculum and posterior middle temporal gyrus (Tranel et al., 2005). The processing of argument structure recruits left IFG (Inferior Frontal Gyrus) including BA47 and BA9, but also the superior temporal (Shetreet et al., 2007), angular and supra-marginal gyri and precuneus, which are more active in processing transitives than intransitives (Den Ouden et al., 2009). Thematic role assignment involves posterior perisylvian areas (Thompson et al., 2007). Tense inflection activates Broca's area, for both regular (e.g., Tyler et al., 2005) and irregular verbs (e.g., de Diego Balaguer et al., 2006). Kielar et al. (2011) report additional involvement of motor, premotor and posterior parietal regions in (overt and covert) present and past tense production. Each of these processes may be selectively impaired when the corresponding neural substrate is damaged, resulting in different sentence production deficits.

### Rehabilitation of verb and sentence production

The interest in the rehabilitation of verb production has increased over the last decades. At the single-word level, verbs can be treated with the same techniques used for nouns, even though improvement in verb production seems more difficult to achieve (Webster and Whitworth, 2012). At the sentence level, treatment techniques typically include identifying the agent and theme of each verb and then producing a sentence including all elements, thereby engaging predicate-argument structure retrieval and thematic role assignment. Aphasic individuals who underwent this type of treatment improved in retrieving treated, but not untreated verbs and showed improvement also in spontaneous speech (Fink et al., 1992; Webster et al., 2005). This suggests that verb production in sentence context may be a more productive treatment strategy than the production of verbs as isolated words. Research with sentence-level treatment has also led to the hypothesis that training complex syntactic structures results in generalization to untrained, linguistically-related, less complex structures (Complexity Account for Treatment Efficacy, Thompson et al., 2003). In line with this hypothesis, treating three-argument verbs in sentence production improved retrieval of untreated one- and two-argument verbs (Thompson et al., 2013).

A linguistically-motivated treatment protocol has been designed to address both lexical-semantic (argument structure) and syntactic (movement) properties of verbs (Treatment of Underlying Forms; Thompson and Shapiro, 2005). This treatment starts by addressing knowledge of/access to the thematic information of verbs. Aphasia patients are subsequently made aware of the properties of movement operations, in an explicit way. The benefits of treatment were shown to generalize to (less complex) constructions requiring the same type of movement as those treated explicitly, and to spontaneous speech (e.g., Thompson et al., 1996), in line with the Complexity Account for Treatment Efficacy (Thompson et al., 2003).

Two studies report on the treatment of verbal morphology by means of a Computerized Visual Communication protocol (Weinrich et al., 1997, 1999). This treatment was used to elicit past, present and future tense forms of regular and irregular verbs in sentences. In both studies, the production of inflected verbs in sentences improved, and generalization was observed in the use of morphological transformations, but not in verb retrieval.

Notably, generalization to lexical retrieval of untreated verbs occurs infrequently (Webster and Whitworth, 2012). The occurrence of generalization may depend on patient characteristics and treatment characteristics. Individuals with semantic damage may be more likely to generalize if treatment restores semantic features that are shared across semantic representations of words. Lexical representations, however, are item-specific and patients with lexical damage are therefore less likely to generalize (Miceli et al., 1996). In what concerns treatment tasks, for nouns, treatments for semantic processing is thought to have greater potential to induce generalization, due to the large overlap of semantic features across words of the same semantic category (e.g., Boyle and Coelho, 1995). However, the same strategy produces only item-specific improvement in verb retrieval (Wambaugh and Ferguson, 2007; Wambaugh et al., 2014).

ACTION is a treatment protocol for aphasia rehabilitation developed for Dutch (Bastiaanse et al., 1997). It includes four steps that address the different levels of processing necessary for producing verbs in simple, declarative sentences:

1. Step 1, lexical level: action naming
2. Step 2, syntactic level: sentence completion with a verb in the infinitive
3. Step 3, morphosyntactic level: sentence completion with finite verb
4. Step 4, sentence construction

In Bastiaanse et al. (2006), treating infinitives did not result in generalization, but treating finite verbs did. Links et al. (2010) found that, when infinitives were treated, untrained infinitives improved only marginally, and untrained finite verbs did not improve. By contrast, when finite verbs were treated, generalization was present for untreated finite verbs, but not for infinitives. Notably, improvement extended to spontaneous speech and to a task tapping communication in daily living, and was sustained after 3 months.

Altogether, the literature shows that when verbs are treated as isolated words, item-specific improvement can be achieved using similar techniques to those used for noun rehabilitation. Generalization to untreated verbs was reported following semantic, gestural and repetition cueing (Rose and Sussmilch, 2008), when treatment was centered at the sentence level and the grammatical properties of verbs were taken into account in designing the treatment task (Bastiaanse et al., 2006; Links et al., 2010; Thompson et al., 2013). These studies share two features—treatment addressed grammatical properties of verbs (e.g., argument structure, inflection, movement) and focused on the sentence level. Engaging knowledge of these abstract properties may be an important ingredient to achieving generalization.

### tDCS in aphasia rehabilitation

Transcranial direct current stimulation (tDCS) is a neuromodulatory technique. A weak electrical current is delivered through electrodes positioned over the scalp (e.g., Nitsche et al., 2008). In language research, studies with healthy individuals show that anodal tDCS can increase speed (Fertonani et al., 2010) and amount of verbal learning (Meinzer et al., 2014). Cathodal tDCS, on the other hand, negatively affected learning in an action and object learning paradigm (Liuzzi et al., 2010). In aphasia rehabilitation, methodology varies substantially across studies. Positive effects were reported in spite of variations in current intensity (1–2 mA), stimulation polarity and montage (perilesional cathodal tDCS in Monti et al., 2008; perilesional anodal in Baker et al., 2010; contralesional cathodal tDCS in Flöel et al., 2011 and contralesional anodal tDCS in Vines et al., 2011) (for reviews, see Hamilton et al., 2011; Monti et al., 2013; de Aguiar et al., 2015).

Models of inter-hemispheric competition (Murase et al., 2004) predict bicephalic montages (a perilesional anode and a contralesional cathode) to modulate interhemispheric interactions more efficiently than monocephalic montages (a perilesional anode and a reference electrode). Recently, it has been suggested that the optimal montage should be determined individually, based on lesion site and size (Hamilton et al., 2011) and the individuals' pattern of activation during correct language production (Baker et al., 2010).

Effective tDCS-related treatment enhancement may depend on appropriately pairing stimulation site and treatment task. Marangolo et al. (2013a, 2014) found that action naming and discourse cohesion were enhanced after stimulation to Broca's but not Wernicke's area^1^. Given that ongoing computations may depend on the pattern of cognitive impairments and brain damage, different patients may respond differently to tDCS. Currently, the lack of data on individual outcomes in many studies (except Marangolo et al., 2013a, 2014), and lack of detailed information about the linguistic deficits of participants do not allow establishing whether some treatments were more effective than others, as a function of lesion site and of cognitive impairment. Supporting the need to report individual outcomes, recent research with healthy participants identified a large variability in individual responses to stimulation (Horvath et al., 2014).

There is little information about the role of tDCS in promoting generalization. Some studies report a transfer to spontaneous speech (Marangolo et al., 2013b, 2014), and statistically insignificant increase of accuracy for untreated nouns (Baker et al., 2010). Nevertheless, in these studies pre-treatment performance was not measured in multiple baselines gathered in a time window similar to that of treatment. In addition, no control task was administered to ensure that behavioral improvement was specific to treatment-related tasks. Therefore, it is not possible to measure the potential effect of task practice nor to rule out spontaneous recovery (Howard et al., 2015). It is relevant to note that generalization could be expected to occur in conversational therapy (Marangolo et al., 2013b, 2014) due to the functional scope of treatment, but it was unlikely in picture-word matching (Baker et al., 2010). To assess the potential of tDCS in enhancing generalization, it is important to pair it with a treatment task likely to yield generalization (e.g., semantic feature analysis for nouns, or linguistically motivated therapies for verbs, such as Treatment of Underlying Forms or ACTION).

As mentioned earlier (Section Rehabilitation of Verb and Sentence Production), generalization in verb production has been observed infrequently. Treatment at the sentence level engaging knowledge of morphosyntactic properties of verbs appears to be effective with this regard, but studies reporting on generalized effects of verb treatment usually focus either on tense training or on argument structure training. In this study, we test the efficacy of the Italian adaptation of the ACTION protocol that combines training of lexical verb retrieval and of verbal morphology, in sentence context. This training should improve lexical retrieval of both treated and untreated verbs. In addition, here we test for the first time whether tDCS, in combination with speech/language therapy, can enhance both item-specific improvement and generalization.

## Method

### Recruitment and participants

The main inclusion criterion was a difficulty in verb retrieval and sentence construction. Eligible participants were nine right-handed^2^ individuals with chronic aphasia after a left hemisphere stroke, aged between 18 and 80 years and with at least 5 years of education. Seven participants presented with their first-ever stroke. The two participants who had had prior lesions were assigned to distinct treatment groups (sham-first and tDCS first). Exclusion criteria were sensitive skin, epileptic seizures in the 6 months preceding enrollment, use of drugs known to increase the risk of seizures and presence of metallic fragments in the head. The study was approved by the ethics committee of the University of Trento (protocol number 2012-035). After being referred by their neurologist, patients and primary caretakers were invited for a briefing session. In this session the procedure was described, and informed written consensus was obtained. Table 1 provides a summary of participants' demographic and clinical characteristics. Detailed information about lesion sites is provided in Supplementary Materials.

**Table 1:** **Demographic and clinical characteristics of participants**.

	**Participant**	**Gender**	**Age**	**Han**.	**Education**	**Occupation**	**Lesion type**	**TPO**
Sham first	LF	M	45	Right	High school	Tinsmith	Ischaemic	39
	GC	M	68	Right	Junior high school	Social worker	Ischaemic	26
	GD	F	48	Right	University degree	Accountant	Hemorrhagic	17
	GP	M	52	Right	High school	Retired	Ischaemic	80
	EC	M	54	Right	High school (incomplete)	Marble worker	Ischaemic	92
tDCS first	SP	M	75	Right	University degree	Accountant	Ischaemic	8
	RL	F	43	Right	High school	Accountant	Ischaemic	88
	CK	F	76	Right	Junior high school	Secretary	Hemorrhagic	54
	PG	M	52	Right	University degree	Insurance actuary	Ischaemic	36

Han., Handedness; TPO, Time Post-Onset (in months).

### Procedure

**Figure 1:**
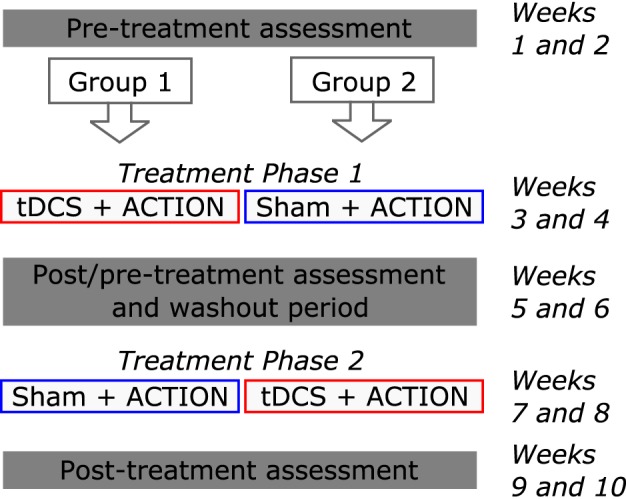
**Treatment study design**. Each patient was involved in two treatment phases. Initially, participants were randomly assigned to one of two treatment orders. The entire protocol lasted 10 weeks.

Prior to the beginning of the experimental protocol, participants were engaged in a diagnostic assessment. A multiple-baseline, double-blind and sham-controlled, cross-over design was used to assess treatment effects. The entire experimental protocol lasted 10 weeks (Figure 1). There were three assessment phases (baseline, intermediate and final), and two treatment phases. In each assessment phase, three testing sessions were spread over a period of 2 weeks, to encompass an interval similar to that of treatment^3^. They served to establish pre-treatment stability in primary outcome and control measures. This allowed to control (unlikely) effects of spontaneous recovery on the changes observed after treatment. In addition, the data from the three sessions that preceded each treatment phase were used to construct two matched sets of verbs: one to be treated, one to measure generalization^4^. The scores obtained in the three pre-treatment assessment sessions were contrasted with those observed in the three post-treatment assessment sessions, to evaluate the effects of treatment on treated and untreated verbs, for each phase. Ten daily (five times per week) 1-h treatment sessions were provided in each treatment phase. Speech-Language Therapy was administered using the Italian version of the ACTION protocol (based on Links et al., 2010), described below. ACTION was administered in both phases, to each individual. Participants were randomly assigned to two possible treatment orders: 5 received Sham in the first, and tDCS in the second treatment phase; 4 received treatment in the reverse order.

#### Diagnostic assessment

A diagnostic language battery (Batteria per l'Analisi dei Deficit Afasici, BADA, Miceli et al., 2006) was administered to identify the functional locus of language impairment. Additional tests for cognitive screening were administered, including Digit Span (Orsini et al., 1987), Clock Drawing (Dal Pan et al., 1989), and Attentive Matrices (Spinnler and Tognoni, 1987).

#### Tests administered in each session of each assessment phase

**Figure 2:**
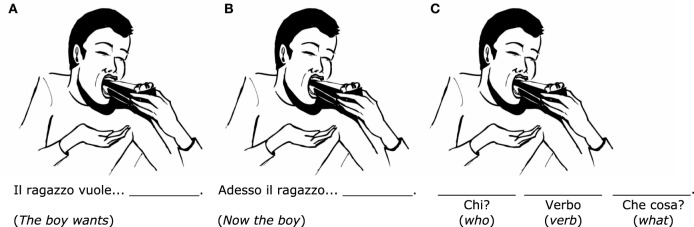
**Examples of stimuli used in the three verb production tests. (A)** VTinfinitive (sentence completion with a verb in the infinitive). **(B)** VTfinite (sentence completion with a verb in the correct tense). **(C)** VTsentence (sentence construction).

Three verb production tests were developed to assess changes in verb retrieval accuracy. Black-and-white line drawings were used to elicit the verb, in all tests (for examples, see Figure 2). In the first task (henceforth, VTinfinitives), participants were asked to complete a sentence (e.g., “L'uomo vuole…,” The man wants…) with the corresponding verb in the infinitive (“…mangiare,” to eat). In the second (henceforth, VTfinite), the to-be-completed sentence included a temporal adverb (e.g., “Ieri/Oggi/Domani l'uomo…,” Yesterday/Now/Tomorrow the man…) and the patient had to produce the finite verb in the correct tense (“…ha mangiato/mangia/mangerà,” ate, eats, will eat). In the third test (henceforth, VTsentence), the patient was prompted with the image, and asked to produce a Subject-Verb-Object (SVO, for transitive verbs, e.g., “L'uomo mangia la torta,” The man eats the pie) or a Subject-Verb-Adjunct (SVA, for intransitive verbs, e.g., “L'uomo corre sulla spiaggia,” The man runs at the beach). The adjunct was always a prepositional phrase expressing location. A complex scoring procedure was developed, but in this report only lexical accuracy is considered—a measure shared by the three verb tests. Responses were scored as correct if the patient produced the correct verb. Phonemic and morphosyntactic errors were disregarded.

The same 88 verbs were used in the three verb production tests. They were divided in three sets (sets 1, 2, and 3). In session 1 of each phase, set 1 was used for VTinfinitive, set 2 for VTfinite and set 3 for VTsentence. In sessions 2 and 3, the three sets were assigned to the three tasks using a Latin Square design. The three sets were matched for relative frequency, length in phonemes, number of internal arguments, instrumentality, name relatedness, body part involved (face, arm, leg), manipulation, inflectional paradigm and regularity. The comparison of lexical accuracy for 88 verbs across the three sessions that preceded each treatment phase allowed establishing pre-treatment stability.

Comprehension of these verbs was assessed using a picture verification test. The picture of a target verb was presented while the examiner pronounced a verb in the infinitive. The verb could be the target, a semantic distractor or an unrelated distractor (e.g., the picture corresponding to the verb “to eat” was paired, on different occasions, to the correct word “to eat,” to the semantic foil “to drink” and to the unrelated foil “to mop”). On each day, 1/3 of the items was presented with the correct target, and the remaining 2/3 with distractors. Participants had to reply “yes” or “no” (verbally or by pressing a key) to indicate whether the verb presented auditorily corresponded to the picture. Targets, semantic and unrelated distractors were matched for frequency, length in phonemes, name relatedness, number of internal arguments, instrumentality and manipulation.

Performance on the nonword repetition test from the BADA (Miceli et al., 2006) was used as a control measure. This allowed assessing whether any observed improvement was treatment-related (i.e., restricted to verb tasks, which were the focus of treatment), or aspecific (nonword repetition measures phonological abilities, but is unrelated to verb retrieval). The test included 36 items, ranging in length between 1 and 3 syllables.

#### Behavioral treatment

In Italian, Subject-Verb-Object is the base word order in sentences. Inflected verbs occur in second position without overt movement. In this study, treatment was provided at the level of simple, declarative sentences, and a task specifically designed to address movement operations was not included. Considering the rich morphology of Italian, steps three and four used in ACTION for Dutch were modified to include verb production in three different tenses. The Italian adaptation of ACTION (Bastiaanse et al., 1997), includes these four steps:

1. Step 1, lexical level: Action naming
2. Step 2, syntactic level: Sentence completion with infinitive
3. Step 3, morphosyntactic level: Sentence completion with finite verb in three tenses
4. Step 4, Sentence construction with finite verb in three tenses

**Figure 3:**
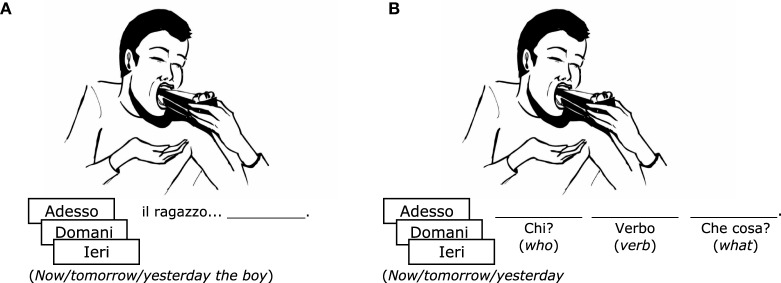
**Examples of stimuli used during treatment. (A)** Step 3 (sentence completion with a verb in three tenses). **(B)** Step 4 (sentence construction in three tenses). Detailed information about cueing procedures for each step is available as Supplementary Materials.

Therapy was provided over ten 1-h sessions in each phase. Each phase lasted 2 weeks and entailed treatment with two different tasks. Participants completed Step 3 in the first week, and Step 4 in the second week. Examples of stimuli for each step are provided in Figure 3 (Figures 3A,B for Steps 3 and 4, respectively). In Step 3, the patient saw an image with an adverb and a subject written below the picture (e.g., “Now the man…”), and was asked to complete the sentence with the verb inflected in the correct tense. In Step 4, the patient saw an image and a written adverb (e.g., “now…”), and was requested to produce a full sentence that properly described the image (Subject-Verb-Object or Subject-Verb-Adjunct), with the verb inflected in the correct tense.

Structured increasing cues were provided. The cues provided to each subject depended on whether the participant produced retrieval errors or morphological errors, and on the constituent in which the error occurred. For a thorough description of the training procedure and of the cueing strategies provided during treatment steps three and four, the reader is referred to the Supplementary Material section. The 88 items included in ACTION were selected on the basis of a norming procedure. Ten healthy volunteers were asked to build sentences that described the picture stimuli. Items with less than 70% picture-sentence agreement across all constituents were excluded based on this data. The items surviving this procedure had a mean agreement of 90.11% (*sd* = 0.084%). In addition, these items were normed for a wide range of linguistic variables to create matched sets of verbs (Rofes et al., 2015).

For each participant, and prior to each treatment phase, two sets of 20 verbs were prepared: a to-be-treated set, to evaluate item-specific benefits of treatment, and a matched, not-to-be-treated set, to evaluate generalization to untreated items. Sets were matched for picture-sentence agreement, age of acquisition, imageability, relative frequency, length in phonemes, number of internal arguments, inflectional paradigm, instrumentality, name relatedness, manipulation, body part involved (face, arm, leg) using available norms (Rofes et al., 2015). In addition, to ensure comparability of treated and untreated items, the two sets were individually tailored. They were matched for retrieval accuracy across the three verb tasks in the three assessments that preceded each treatment phase, for error types produced by the patient, and for comprehension accuracy^5^. The details of set balancing for each patient are available as Supplementary Material.

#### tDCS

tDCS was administered using a battery-driven, programmable Eldith direct current stimulator (neuroConn, PLUS version), through two 35 cm^2^ electrodes. Current intensity was increased in a ramp-like fashion for 5 s until reaching 1 mA (current density = 0.2 mA/cm^2^). Each treatment session began with 20 min of real or sham bicephalic tDCS. Sham stimulation was administered with the same parameters used for real stimulation, but the stimulator was turned off after 30 s (Gandiga et al., 2006). The same procedure was repeated at the end of the 20-min period. To ensure blinding efficiency, participants were asked to fill a questionnaire at the end of each 2-week treatment phase (Fertonani et al., 2010), in which they indicated the nature and intensity of the sensations experienced during the treatment. Participants reported mild to moderate itchiness, pinching, burning, fatigue or heating under the electrode, mild pain. One patient reported mild headache and two others reported mild discomfort under the elastic strap.

Both the therapist who administered behavioral treatment and the experimenter who analyzed the data were blind to the stimulation condition, until individual outcomes for phase 1 and 2 were statistically analyzed. A third experimenter handled the tDCS device in each treatment session. The difference in number and intensity of symptoms observed across tDCS and Sham phases was not significant (Wilcoxon Signed-Rank test = 0.84, *p* = 0.200, one tailed).

Stimulation site was determined individually, after inspection of each patient's MRI scan (see Table 2). The anode was always centered over a left perilesional area. In three participants (CK, PG and GD), this was Broca's area (BA 44–45), and in these cases the cathode was placed over the right hemisphere homolog of Broca's area. In two cases (LF and EC) the lesion partially encompassed Broca's area. In these subjects the anode was placed anterior and superior to Broca's area (BA45–46), and the cathode over the homologous area in the right hemisphere. In three other participants (GP, RL and SP) lesions were more anterior. Since they encompassed the entire IFG and caused considerable damage to the middle frontal gyrus, the anode was placed over the left superior and middle frontal gyri (BA9–10). In these cases, the cathode could not be positioned symmetrically, because shunting of current between electrodes (bypassing the brain) can occur with electrode distances under 8 cm (DaSilva et al., 2011). Therefore, the cathode was positioned over the right homolog of Broca's area. Finally, GC's lesion was parieto-occipital and parieto-temporal. In order to respect the rule of stimulating peri-lesional areas, the anode was positioned over the posterior middle and superior temporal gyri (encompassing Wernicke's area), and the cathode in a symmetrical position over the RH.

**Table 2:** **Stimulation sites and electrode positioning**.

	**Anode (LH)**	**EEG coordinates**	**Cathode (RH)**
LF	Anterior and superior to Broca's area (BA45-46)	Centered between F7 and F3	Homologous
GC	Superior/middle temporal gyri (BA21-22)	Centered between T7 and TP7	Homologous
GD	Broca's area (BA 44–45)	Crossing point between T3-Fz and F7-Cz	Homologous
GP	Superior/middle frontal gyri	Centered above FP1	Right Broca
EC	Anterior and superior to Broca's area (BA45-46)	Centered between F7 and F3	Homologous
SP	Superior/middle frontal gyri (BA10)	Centered above FP1	Right Broca
RL	Superior/middle frontal gyri (BA10)	Centered above FP1	Right Broca
KC	Broca's area (BA 44-45)	Crossing point between T3-Fz and F7-Cz	Homologous
PG	Broca's area (BA 44-45)	Crossing point between T3-Fz and F7-Cz	Homologous

EEG coordinates are expressed according to the international 10–20 system. LH, left hemisphere; RH, right hemisphere.

Broca's area was identified as the crossing point between T3-Fz and F7-Cz, following Friederici et al. (1998). All other coordinates were extracted from Okamoto et al. (2004), who studied the probabilistic mapping of 10-20 EEG coordinates and brain areas on the cortical surface.

## Results

### Diagnostic assessment

Selected tests from the BADA (Miceli et al., 2006) were used to characterize the profile of language impairment in each subject. Results of this diagnostic assessment are presented in Table 3. Our sample included fluent (GC, GD, and PG) and nonfluent participants. In all cases, sentence production was characterized by omission of obligatory arguments, errors of thematic role assignment, morphological errors and difficulties in producing noncanonical sentences. Three participants had mild-to-moderate semantic impairment (GD, SP, and KC). All participants presented with damage to the phonological output lexicon. Different sublexical conversion mechanisms were impaired across subjects, but these always included phoneme-to-phoneme conversion. In addition, all participants presented with length-sensitive difficulties in tasks that required overt production, suggesting damage to phonological short-term memory. The diagnostic assessment of each patient is summarized in the Supplementary Materials.

**Table 3:** **Scores (% error) in diagnostic assessment battery (BADA)**.

		**Sham first**					**tDCS first**			
		**LF**	**GC**	**GD**	**GP**	**EC**	**SP**	**RL**	**CK**	**PG**
Sublexical	Auditory discrimination	6.7	6.7	6.7	10.0	3.3	n.a.	10.0	10.0	3.3
	Visual-auditory discrimination	13.3	0.0	13.3	43.3	11.7	n.a.	20.0	n.a.	n.a.
	Nonword repetition	27.8	26.1	33.3	27.8	27.8	44.4	5.6	55.6	27.8
	Nonword reading	26.1	22.7	0.0	80.0	35.6	91.3	21.7	30.4	47.8
	Nonword writing	66.7	61.5	8.3	100.0	72.0	n.a.	41.7	75.0	41.7
	Nonword copy	50.0	n.a.	0.0	0.0	33.3	n.a.	0.0	16.7	16.7
Semantico-lexical	Auditory lexical decision	12.5	10.0	12.5	11.3	8.8	8.8	5.0	22.5	10.0
	Visual lexical decision	30.0	7.5	0.0	31.3	7.5	37.5	0.0	7.5	17.5
	Word repetition	27.3	0.0	4.5	2.2	2.2	55.6	9.1	4.5	27.3
	Word reading (aloud)	32.6	2.2	2.2	56.5	0.0	60.9	4.3	0.0	30.4
	Word writing to dictation	69.6	8.7	0.0	80.0	80.4	n.a.	8.7	17.4	17.4
	Word copy	40.0	20.0	0.0	20.0	40.0	100.0	0.0	0.0	40.0
	Auditory noun comprehension	10.0	2.5	20.0	2.5	0.0	30.0	0.0	5.0	0.0
	Visual noun comprehension	10.0	0.0	0.0	0.0	0.0	10.0	0.0	10.0	0.0
	Auditory verb comprehension	10.0	0.0	0.0	0.0	0.0	10.0	0.0	40.0	0.0
	Visual verb comprehension	10.0	5.0	0.0	30.0	0.0	40.0	0.0	30.0	0.0
	Oral object naming	66.7	20.0	60.0	16.7	43.3	60.0	0.0	13.3	33.3
	Written object naming	63.6	9.1	45.5	50.0	95.5	n.a.	27.3	18.2	36.4
	Oral action naming	71.4	28.6	57.1	78.6	57.1	64.3	0.0	14.3	42.9
	Written action naming	90.9	9.1	81.8	90.9	100.0	n.a.	27.3	36.4	54.5
Grammatical	Picture description—unconstrained	100.0	25.0	75.0	100.0	50.0	100.0	n.a.	100.0	100.0
	Picture description—constrained	100.0	70.0	80.0	100.0	80.0	100.0	n.a.	100.0	100.0
	Sentence repetition	40.0	30.0	10.0	60.0	5.0	n.a.	20.0	50.0	50.0
	Sentence reading	66.7	n.a.	0.0	100.0	0.0	n.a.	33.3	0.0	66.7
	Auditory comprehension	15.0	8.3	10.0	28.3	11.7	92.3	3.3	13.3	13.3
	Visual comprehension	26.1	3.3	13.0	26.7	4.4	n.a.	0.0	26.1	26.1

Underlined scores fall below norm.

### Cognitive screening

LF, GP, SP, RL, CK, and PG performed below norm in the forward Digit Span, consistent with reduced phonological short-term memory. All participants except RL performed below norm in digit span backwards. SP, KC and PG did not complete this task. Visual attention, as assessed by Attentive Matrices, was impaired in LF, GC, EC, SP, and RL. Visuo-spatial cognition and two-dimensional construction, as assessed by the Clock-Drawing test, was below norm in LF, EC, SP, and RL. Subject GD did not complete these two tasks, due to difficulties following instructions. Scores for each participant are presented in Table 4.

**Table 4:** **Scores in cognitive screening tasks**.

		**Cut-off**	**Sham first**					**tDCS first**			
			**LF**	**GC**	**GD**	**GP**	**EC**	**SP**	**RL**	**CK**	**PG**
STM and WM^1^	Forwards (0–8)	3.75	3.5	4.3	5.3	1.8	4.0	3.5	2.5	3.3	3.3
	Backwards (0–8)	5 ± 2	2.0	2.0	2.0	1.8	2.0	n.a.	4.0	n.a.	n.a.
Visual attention		31	27.3	23.0	n.a.	46.0	16.0	12.3	15.8	45.5	28.3
Visual-spatial cognition and two-dimensional construction		v.n. > 3	3.0	4.0	n.a.	5.0	3.0	-1.0	3.0	10.5	12.0

Digit Span (Orsini et al., 1987); Attentive matrices (Spinnler and Tognoni, 1987); Clock Drawing Test (Dal Pan et al., 1989). Underlined scores fall below norm.

### Group results

#### Treatment effects: lexical accuracy in verb production

**Figure 4:**
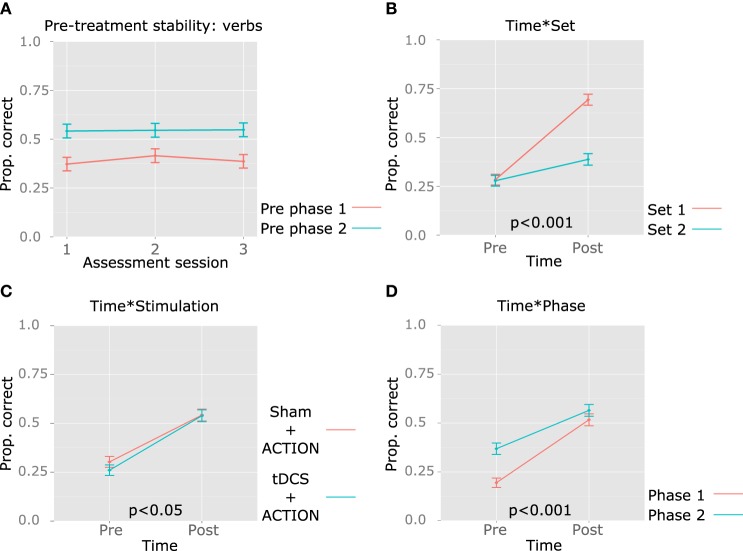
**Group results: pre-treatment stability and effects of treatment in verb retrieval**. The mean proportion of correct responses is represented in the y axis. **(A)** Stability in performance in the three sessions that preceded each therapy phase (no significant differences observed for either phase). **(B)** Time^*^Set interaction. **(C)** Time^*^Stimulation interaction. **(D)** Time^*^Phase interaction. The *p*-value for each significant interaction is reported above the x axis.

Group data were analyzed by computing a generalized linear mixed model for logistic data, using model comparison to assess the need to include each factor (Jaeger, 2008). Models were computed using R package lme4 (Bates et al., 2014). The dependent variable was accuracy in verb production. Responses were scored as accurate if the target verb was produced, disregarding phonemic paraphasias and morphosyntactic errors. Pre-treatment stability was established by comparing accuracy between the three sessions that preceded each treatment phase, including all 88 verbs. For pre-treatment stability, the null model included random intercepts for Participants and Items. We tested this model against a model containing fixed effects for Session (assessment sessions 1, 2, and 3 prior to each treatment phase). The alternative model did not provide a better fit for the data in comparison to the null model in either phase 1 [χ^2^(1) = 0.3512, *p* = 0.5534] or phase 2 [χ^2^(1) = 0.0708, *p* = 0.7902], and the main effect of Session fell very far from significance (phase 1: *z* = 0.593, *p* = 0.5530; phase 2: *z* = 0.266, *p* = 0.790), showing stable behavior for this group of participants, before each treatment was administered (see Figure 4A).

**Figure 5:**
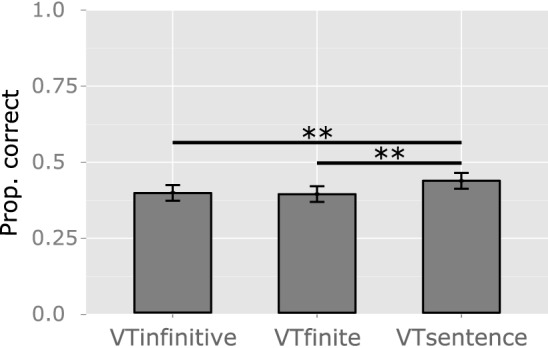
**Group results: differences in performance across the three verb tests**. The y axis represents the mean proportion of correct responses (Prop. correct). VTinfinitive: sentence completion with a verb in the infinitive. VTfinite: sentence completion with a verb in the correct tense. VTsentence: sentence construction. Significant results are reported: ^**^p < 0.01.

Treatment outcome was established by computing a second model. The model included random intercepts for participants, with random slopes for Set^*^Time (Set = treated, untreated; Time = pre-, post-treatment), as patients may respond differently to treatment and show different degrees of generalization. Since differences are expected only in the post-treatment assessment, an interaction was relevant. We also included random intercepts for items, with random slopes for Set, because differences between the treated and the untreated set may vary between items. The model improved significantly with the main effects of Time (pre-, post-treatment), Set (treated, untreated verbs), Phase (1 and 2), Stimulation (Sham, tDCS) and Verb Test (VTinfinitive, VTfinite, VTsentence), and the interactions Time^*^Set, Time^*^Phase, Time^*^Stimulation. Figures 4, 5 illustrate the relevant main effects and interactions, and corresponding statistics are reported in Table 5. *Post-hoc* pairwise comparisons were computed to characterize the main effect of VerbTest and significant interactions. For this purpose, we used the lsmeans package in R (Lenth and Hervé, 2015), and selected the Scheffe method for adjusting *p*-values for multiple comparisons.

**Table 5:** **Summary of fixed effects (verb accuracy)**.

	**Estimate**	**Std. Error**	***z*-Value**	**Pr(>|z|)**
(Intercept)	−1.5163	0.30917	−4.904	9.38E-07^***^
Time (pre vs. post)	2.58453	0.32153	8.038	9.11E-16^***^
Set (treated vs. untreated)	−0.0495	0.15385	−0.321	0.74787
Phase (1 vs. 2)	0.97611	0.11365	8.589	<2E-16^***^
Stimulation (Sham vs. tDCS)	−0.3583	0.11301	−3.17	0.00152^**^
VerbTest (VTinfinitive vs. VTfinite)	−0.0252	0.091	−0.276	0.78223
(VTinfinitive vs. Vtsentence)	0.23501	0.09038	2.6	0.00932^**^
Time^*^Set	−1.6836	0.34451	−4.887	1.02E-06^***^
Time^*^Phase	−0.7923	0.152	−5.212	1.86E-07^***^
Time^*^Stimulation	0.33311	0.15142	2.2	0.02781^*^

Formula: glmer(Accuracy ~ Time^*^Set + Time^*^Phase + Time^*^Stimulation + VerbTest + (1+Set^*^Time|Participant) + (1+Set|Item), data, family = “binomial”).

Significant results are reported:

*** p < 0.001;

** p < 0.01;

* p < 0.05.

The significant main effect of Time reflected the efficacy of the treatment provided across two phases (ACTION + tDCS or ACTION + Sham), for both treated and untreated verbs. No main effect of Set was observed, as treated and untreated verbs were matched in baseline accuracy. However, the interaction Time^*^Set was significant (Figure 4B), showing greater improvement for treated verbs. *Post-hoc* tests confirm that the lack of differences between verb sets before treatment (*p* > 0.9), but after treatment patients responded more accurately to treated verbs (*z* = 4.709, *p* = 0.0001), and between the two assessments, accuracy improved significantly for both treated (*z* = 7.713, *p* < 0.0001) and untreated verbs (*z* = 5.175, *p* < 0.0001). A main effect of stimulation indicates that scores in the tDCS phase were lower than those collected in the Sham phase, and the interaction Time^*^Stimulation denotes greater improvement in the real tDCS condition. *Post-hoc* tests clarify that improvement was significant both in the Sham (*z* = 7.686, *p* < 0.0001) and tDCS phases (*z* = 9.467, *p* < 0.0001), and while pre-treatment accuracy was lower in the tDCS condition (*z* = −3.170, *p* = 0.018), differences between tDCS and Sham are not significant after treatment (*p* > 0.9) (Figure 4C).

Scores observed in Phase 2 were higher than those observed in Phase 1, as shown by the main effect of Phase. The interaction Time^*^Phase indicates that the amount of improvement was smaller in Phase 2 (Figure 4D). In *post-hoc* tests, scores were higher in Phase 2 in comparison to Phase 1 before (*z* = 8.589, *p* < 0.0001) but not after treatment (*z* = 1.708, *p* = 0.404), and significant improvement was observed both in Phase 1 (*z* = 10.631, *p* < 0.0001) and in Phase 2 (*z* = 6.448, *p* < 0.0001). Patients fared better in VTsentence, than in VTinfinitive (*z* = 2.600, *p* = 0.034) and VTfinite (*z* = 2.875, *p* = 0.016), but differences in accuracy between VTinfinitive and VTfinite and the interaction with Time fell short of significance (*p* > 0.9 and *p* > 0.4, respectively) (Figure 5).

#### Control task: nonword repetition

**Figure 6:**
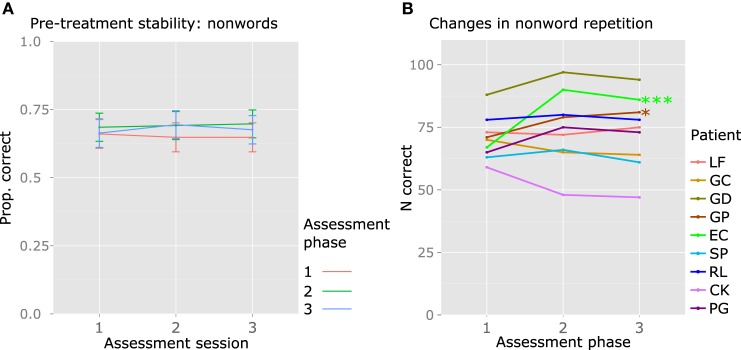
**Group and individual results: performance in the control task nonword repetition. (A)** Group data; the y axis represents the mean proportion of correct responses (Prop. correct), across the three sessions of each assessment phase, and the lines represent the different assessment phases (before treatment 1, after treatment 1, after treatment 2). **(B)** Individual data; the y axis represents the number of correct responses (N correct) across the three sessions of each assessment phase (max. 108). Significant results are reported: ^***^p < 0.001; ^*^p < 0.05. For EC and GP, a significant increase in nonword repetition accuracy was observed between the first and the second assessment phases.

Aspecific improvement was assessed with a nonword repetition task, administered in the three sessions of each assessment phase. Significant changes between assessments 1 and 2, and/or 2 and 3, would indicate aspecific improvement. The null model included random intercepts for Patient and Item. An alternative model introducing random slopes for Time, under the assumption that different patients may present different degrees of aspecific improvement, was the only model that significantly improved fit [χ^2^(5) = 23.673, *p* = 0.0003]. This suggests that some participants may show improvement in nonword repetition. Main effects of Assessment phase (1, 2, and 3), Assessment Day (1,2,3, within each phase), and their interaction, did not improve model fit. At the group level, nonword repetition was stable within and between assessments (see Figure 6).

### Individual outcomes

#### Treatment effects: lexical accuracy in verb production

**Figure 7:**
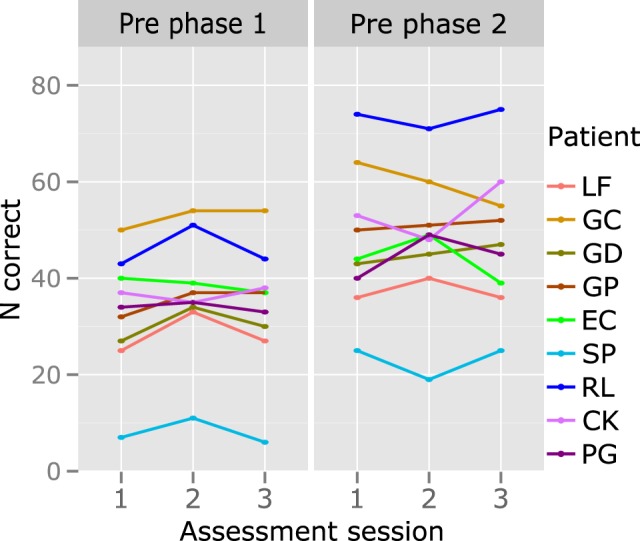
**Individual results: behavioral stability prior to each treatment phase**. The y axis represents the number of correctly produced verbs (N correct; max. 88 in each of the three sessions that preceded treatment phases 1 and 2). No significant changes are observed.

For each participant, baseline stability was checked before each treatment phase by comparing lexical accuracy in the three sessions preceding treatment, by means of Cochran's Q-test. All participants presented stable behavior prior to each phase (see Figure 7).

Significant improvements between the pre- and post-assessments were computed for each treatment phase, for treated and untreated verbs. Given that each verb had been produced three times in the three sessions of pre- and post-therapy assessments, verb retrieval accuracy scores were calculated by collapsing across performance on the three administrations, thus reaching a final 3-point outcome measure of 3-day lexical accuracy. This procedure has been used to increase score sensitivity (Flöel et al., 2011). Differences between pre- and post-therapy assessments were tested using the Wilcoxon Signed-Rank Test.

**Figure 8:**
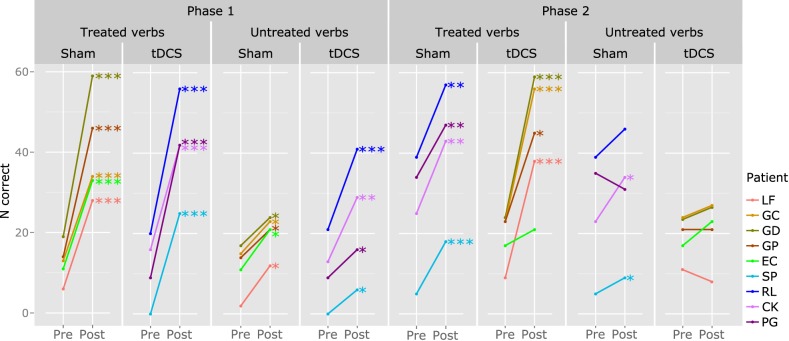
**Individual results: effects of treatment in verb retrieval**. The y axis represents the number of correct responses (N correct; max. 60, corresponding to 20 verbs over 3 tests). This information is available for treated (left) and untreated verbs (right), for each treatment phase and stimulation condition. Significant results are reported: ^***^p < 0.001; ^**^p < 0.01; ^*^p < 0.05.

Significant improvement of treated verbs was observed in all participants, in both stimulation conditions, except for EC in the real tDCS condition (coinciding with Phase 2) (see Table 6 and Figure 8). The extent of item-specific improvement in each phase was compared using Fisher exact tests. In EC, improvement was significantly greater in the Sham phase, as compared to the tDCS phase (Fisher exact *z* = 3.5319, *p* = 0.0002). Item-specific improvement across phases did not differ significantly in the other participants.

**Table 6:** **Individual treatment outcomes for treated and untreated verbs**.

**Participant**	**ACTION+**	**Phase**	**Treated verbs**				**Untreated Verbs**			
			**Pre**	**Post**	***V***	***p***	**Pre**	**Post**	***V***	***p***
LF	Sham	1	6	28	0.000	0.000	2	12	4.000	0.012
	tDCS	2	9	38	0.000	0.000	11	8	20.000	0.890
GC	Sham	1	13	34	0.000	0.000	15	23	19.500	0.025
	tDCS	2	23	56	0.000	0.000	24	27	27.000	0.307
GD	Sham	1	19	59	0.000	0.000	17	24	8.000	0.040
	tDCS	2	24	59	0.000	0.000	24	27	9.000	0.215
GP	Sham	1	14	46	0.000	0.000	14	21	15.000	0.049
	tDCS	2	23	45	10.000	0.001	21	21	33.000	0.519
EC	Sham	1	11	33	5.000	0.000	11	21	9.000	0.014
	tDCS	2	17	21	32.500	0.168	17	23	16.500	0.060
SP	Sham	2	5	18	0.000	0.006	5	9	4.000	0.205
	tDCS	1	0	25	0.000	0.000	0	6	0.000	0.047
RL	Sham	2	39	57	0.000	0.002	39	46	9.000	0.048
	tDCS	1	20	56	0.000	0.000	21	41	7.000	0.000
CK	Sham	2	25	43	0.000	0.002	23	34	18.000	0.012
	tDCS	1	16	42	0.000	0.000	13	29	9.000	0.005
PG	Sham	2	34	47	5.500	0.003	35	31	42.000	0.811
	tDCS	1	9	42	0.000	0.000	9	16	15.000	0.049

Pre- and Post-scores are expressed by the total number of correct responses in each assessment phase (max = 60). Pre- and post-treatment scores were compared by the Wilcoxon Signed-Rank Test.

The same procedure was used to assess generalization (improved production of untrained verbs). LF, GC, GD, GP, and EC improved significantly on untreated verbs in the Sham condition (coinciding with Phase 1), but not in the tDCS condition (Phase 2). SP and PG presented significant generalization in the tDCS phase (coinciding with Phase 1), but not in the Sham phase (Phase 2). RL and KC had significant generalization in both phases. The amount of generalization was significantly higher in the tDCS phase for PG (Fisher exact *p* < 0.001) and in the Sham phase for LF (Fisher exact *z* = 4.4563, *p* = 0.0000) and GP (Fisher exact *z* = 2.1354, *p* = 0.0000).

#### Control task: nonword repetition

Nonword repetition scores during the three assessment phases were contrasted, to determine stability prior to each treatment phase. Performance was stable in all participants, except GC, before phase 2 [Cochran's Q-test (2) = 6.222, *p* = 0.0446]. In this subject, nonword accuracy increased significantly between sessions 1 and 2 of the assessment phase that preceded treatment phase 2 [McNemar's χ^2^test (2) = 4.1667, *p* = 0.0412], but did not increase further in the third session. We have no clear account for this observation, as session 3 was not significantly different from either session 1 (*p* > 0.2) or session 2 (*p* = 0.6).

Following the procedure used for verbs, the sum total of the correct responses produced during the three sessions of each assessment phase was calculated, to obtain a 3-point measurement of nonword repetition accuracy for each assessment in each participant. The comparison of this measure across assessments 1 (before phase 1), 2 (after phase 1 and before phase 2), and 3 (after phase 2), allowed to measure aspecific improvement in each participant. GP [Friedman's test χ^2^(2) = 6.889, *p* = 0.0319] and EC [Friedman's test χ^2^(2) = 19.4783, *p* < 0.0001] showed significantly increased accuracy in the second assessment compared to the first, that is, after Sham (treatment phase 1) (GP: Wilcoxon Signed-Rank test = 2.5, *p* = 0.0282; EC: Wilcoxon Signed-Rank test = 0, *p* = 0.0007). Neither patient's accuracy increased further in the third assessment (Figure 6).

## Discussion

In this study, we found that patients had a stable performance accuracy across the three sessions that preceded each treatment phase. Analyses of pre- and post-treatment data revealed main effects of Time, Phase, Stimulation, and Verb Test. The interactions Time^*^Phase, Time^*^Set, and Time^*^Stimulation were significant. Performance in the control task (nonword repetition) was stable across assessments. Baseline stability and lack of significant changes in a control task allow to attribute the observed changes to therapy (Nickels et al., 2015). Overall, we observe better verb retrieval in sentence construction than in the other two verb tests. In addition, significant improvement is observed for both treated and untreated verbs. The amount of improvement is larger for treated verbs, in Phase 1, and in the real tDCS phase. Individually, all patients showed both item specific improvement and generalization, to different degrees across phases and stimulation conditions. In the following section we discuss the nature of treatment effects and the potential contribution of tDCS to these effects.

### Item-specific effects and generalization with ACTION

Speech/Language Therapy (ACTION ± tDCS) effectively increased response accuracy, and this improvement was statistically significant for both treated and untreated verbs, at the group level. Albeit present for both sets, improvement was larger for treated verbs. This outcome was expected, as other studies have shown the efficacy of treating verb production in sentences (Edwards and Tucker, 2006), in particular when knowledge of predicate-argument structure is trained explicitly (Fink et al., 1992; Webster et al., 2005; Thompson et al., 2013). Semantic (Edwards and Tucker, 2006), phonemic (e.g., Fink et al., 1992), written word (Conroy et al., 2009), and repetition cues (e.g., Weinrich et al., 1999) all improved retrieval of treated verbs. Indeed, the verbs included in ACTION-based treatment improved in every phase of therapy in all subjects, except for EC, who improved only in Phase 1.

Comparable pre-treatment accuracy across the two sets is essential to identify generalization. Post-treatment accuracy improved significantly for both sets at the group level. In addition, significant generalization occurred in individual cases. It was present in 9/9 participants, either in the first phase (9/9) or in both phases (2/9). ACTION treatment yielded generalization in Dutch and German individuals with aphasia (Bastiaanse et al., 2006; Links et al., 2010). Its Italian adaptation, that adds a specific focus on verb morphology, further encourages the adoption of a structured cueing hierarchy in order to provide patients with a strategy conducive to both item-specific and generalized improvement.

Stable nonword repetition performance at the group level suggests that improvement of verb retrieval was due to treatment, and not to task practice (Nickels et al., 2015). The same holds at the individual level, except in EC and GP, whose nonword repetition accuracy improved in the same phase in which generalization occurred. Prior to participating in this study, EC had not received Speech-Language Therapy for 4 years, and GC had followed (not during his participation in this study) a treatment protocol that also included repetition tasks. For these two cases, improvement in an untreated task does not allow to establish the reasons for better performance on untrained verbs in experimental tasks—it could be attributed to treatment, but also to a charm effect or to the adoption of strategies external to ACTION. Nevertheless, since in both subjects performance in additional tasks (e.g., object naming) was stable throughout the protocol, and since in the other participants nonword repetition did not improve, it is reasonable to attribute generalization to ACTION, at least in part, also in the case of EC and GP.

Which mechanisms may have resulted on generalization? The representation of a verb specifies, in addition to suprasegmental and syllabic/segmental features (represented also for nouns), lexical-grammatical properties that are exclusive to verbs, such as conjugation, inflectional paradigm, transitivity, predicate-argument structure, etc.). Such properties are verb-specific, but are similar for large sets of items. In fact, there is evidence that different verbs share information about the syntactic structures in which they occur (Pickering and Branigan, 1998), and that this can result in structural priming between sentences that include different verbs (Bock, 1986). Consequently, training predicate-argument structure production in the context of a specific verb can facilitate retrieval of the same predicate-argument structure for another verb. And in turn, it can facilitate activation of lexical items that are semantically appropriate to the active predicate-argument structure (Bock, 1986). This lexical selection bias can enhance access to the representations of untreated verbs. In short, participants might have benefited from improved retrieval of treated verbs, and from recovered knowledge of typical argument structure to cue the retrieval of untreated verbs. At the end of the treatment protocol, this might have yielded both item-specific recovery and generalization.

Interestingly, generalization was observed in protocols that require production of verbs in sentence context (Bastiaanse et al., 2006; Links et al., 2010; Thompson et al., 2013), but not in protocols focusing on verb production at the single-word level, even when action naming was preceded by explicit discussion of that verb's argument structure (e.g., with modified semantic feature analysis for verbs; Wambaugh and Ferguson, 2007). This suggests that generalization depends not only on training lexical verb retrieval or on recovering abstract knowledge of argument structure, but also on actually producing predicate argument structures.

The role of structural complexity should also be considered here. In the second week of each therapy phase, the treatment task reached a higher level of complexity than that used in any of the tasks used during assessment. At this stage, participants were prompted with an image and an adverb and were asked to produce full sentences with verbs inflected in the correct tense. Even the most demanding task used to measure improvement (sentence construction) was simpler than this treatment task in some respects, as participants need not inflect the verb in one of three tenses. Importantly, all tasks tackled related linguistic operations. The Complexity Account for Treatment Efficacy predicts improvement in linguistically related, less complex tasks (Thompson et al., 2003). Improved verb retrieval for untreated verbs in less complex, related structures, was also reported (Thompson et al., 2013), with 3-argument verb treatment resulting in improved production of 1- and 2-argument verbs in sentences. In addition, morphosyntactic complexity was shown to have an impact in verb retrieval, with aphasic patients displaying poorer retrieval of finite than nonfinite verbs (Bastiaanse, 2011). By treating the production of tense morphology (a knowledge that can be generalized), we may have decreased task complexity for both treated and untreated verbs, thereby allowing resource allocation for lexical selection processes.

In most participants, difficulties in sentence construction were associated with damage to multiple levels of language processing, including semantics, lexical retrieval, sublexical conversion procedures, working memory and grammar (thematic role assignment, realization of predicate-argument structure, and morphosyntactic processes). Focusing treatment on verb retrieval, verbal morphology and predicate-argument structure in sentence-level tasks may have indirectly yielded additional benefits (generalization) by alleviating associated impairments and/or implicitly teaching participants how to circumvent them. For example, training may have increased working memory capacity, and the improvement of grammatical processing may have decreased the cognitive load associated with sentence construction, resulting in more efficient allocation of resources to lexical retrieval.

Given that verb accuracy was calculated by collapsing accuracy across three different tasks, we also considered whether this scoring procedure influenced the evaluation of performance and the resulting patterns of improvement. There was a main effect of Verb Test, indicating that participants retrieved verbs more accurately in the VTsentence (sentence construction) than in the other two tasks, possibly because in this task patients read cues about the nature of the constituents to produce (see Figure 2), and this may have facilitated access to predicate-argument structure. Patient also had more time to respond in this task (30 s, in comparison to 20 s in the other tasks), to account for the higher number of words that needed to be produced. Importantly, after therapy, lexical verb retrieval improved in all tests (VTinfinitive, VTfinite, VTsentence), without significant across-task differences.

Since participants were treated in two phases, and were randomly assigned to the two stimulation sequences (tDCS, then sham vs. sham, then tDCS), the effect of timing on treatment is worth considering. Participants improved more in Phase 1 than in Phase 2. This may have occurred because there was more room for improvement in Phase 1 (subjects had not received any treatment for several months), and recovery plateaued by the end of Phase 2. Following TUF-based treatment (Thompson and Shapiro, 2005), Dickey and Yoo (2013) showed that improvement of treated and untreated verbs depends on different dose-response relations. Treated verbs were acquired faster and linearly, whereas generalization emerged more slowly, its learning curve accelerating over time. In the present study, both item-specific improvement and generalization were larger in Phase 1, and the pattern for untreated verbs was opposite to that reported by Dickey and Yoo (2013).

### tDCS

Scores before and after the tDCS treatment phase were lower than those before and after the Sham phase, as shown by the main effect of Stimulation. In fact, we successfully controlled pre-treatment accuracy across treated and untreated verbs in each phase, but accuracy across phases was more difficult to balance, as it depended on the extent to which each participant improved in Phase 1. The Time^*^Stimulation interaction suggests that, in spite of lower initial scores, improvement was greater in the tDCS phase. However, this result must be taken cautiously, as the steeper slope for real tDCS may reflect a true enhancement due to successful neuromodulation, but also a ceiling effect for the Sham condition. In other words, if participants could not improve further than observed, the slope may be steeper in the tDCS condition just because participants started off with lower accuracy. We discuss these possibilities (a true stimulation effect and a ceiling effect) in the next paragraphs.

To our knowledge, this is the first time that tDCS is applied together with a treatment program that targets verb production in sentence context and includes explicit morphosyntactic training. Neuroimaging studies suggest that sentence production and verb inflection require computations that are widely distributed in the brain (e.g., Perani et al., 1999; Thompson et al., 2007). Given that tDCS is more effective when the electrodes are placed directly above areas involved in the cognitive processes associated with stimulation (Marangolo et al., 2013a), it is possible that tDCS is more effective when associated with cognitive functions that have a more circumscribed representation. Thus, ACTION could be considered a less optimal protocol to pair with tDCS. Nevertheless, previous research contradicts the idea that widespread representation of the cognitive processes engaged by a task may decrease efficacy of neuromodulation. For example, benefits from tDCS were reported in association with conversational therapy (Marangolo et al., 2013b).

Stimulation was delivered to different sites in different participants. We did this to ensure that tDCS was applied over healthy tissue in each case. In previous research (Baker et al., 2010), stimulation sites were identified based on each individual's fMRI activation during correct naming. This procedure was selected to ensure that the stimulated area was involved in the to-be-treated task, and to putatively allow tDCS to enhance patterns of activation known to correlate with good performance. While this approach has pragmatic limitations (discussed in de Aguiar et al., 2015), it is indeed relevant to target areas for stimulation that have at least the potential to be involved in the task. Our decision in terms of stimulation site may have resulted in a more efficient pairing of functional role of the area and treatment task in some cases than in others (see Marangolo et al., 2013a), but this approach was preferred to stimulation of lesioned tissue. First, because lesioned tissue can disturb current flow (Datta et al., 2011) and, most importantly, because recovery is typically associated with activation of peri-lesional or contra-lesional areas (Schlaug et al., 2008) and tDCS directly over lesioned areas was reported to be ineffective (Hesse et al., 2007).

Individual data analyses highlight another important issue. For treated verbs, EC had larger improvement in the Sham condition. For untreated verbs, improvement was greater after tDCS for PG, and after Sham for LF and PG. Crucially, these participants showed greater improvement in Phase 1 than in Phase 2, regardless of stimulation condition. The same was true at the group level. Therefore, it is not clear whether across-phase differences are due to type of stimulation (tDCS vs. Sham) or to treatment phase (1 vs. 2). In cross-over designs, in which typically two treatments are administered over two phases, treatment order can massively influence outcome. In our sample, five participants received Sham first and four received tDCS first. With an uneven number of subjects, and a significantly larger improvement in Phase 1, the design is somewhat biased toward larger improvements in the Sham condition. Nonetheless, group analyses show greater improvement in the tDCS phase, for both treated and untreated verbs.

All things considered, in the same way that we cannot rule out a ceiling effect for Sham, we can also not exclude the possibility that data reflect a true, tDCS-related enhancement. Assuming a real effect of tDCS, our data is in line with previous research. Performance in tasks using verbs, such as action naming (Marangolo et al., 2013a) and spontaneous speech (Marangolo et al., 2013b, 2014), showed significant therapy enhancement after stimulation of Broca's area. In our study, the anode was placed over Broca's area in three participants and over the neighboring left hemisphere cortex in five. Considering that we focused on verb retrieval accuracy, our data are consistent with those of Marangolo et al. (2013a), showing that stimulation of Broca's area (and of the surrounding cortex)^6^ can enhance verb production. Since a bi-cephalic montage was used in all participants, the observed effects could be due to a combination of the excitation induced by the anode placed over LH perilesional areas, and of the active role of the cathode over contralesional areas (Nitsche et al., 2008), which may have contributed to balancing interhemispheric competition (Murase et al., 2004).

In addition, lack of a three-way interaction involving Set (Time^*^Stimulation^*^Set) suggests that greater improvement in the tDCS phase involves both treated and untreated verbs. Moreover, control for aspecific improvement in verb production was achieved (pre-treatment performance was stable, and no group-level effects were observed for nonwords), and therefore data indicate that improvement of untreated items reflects generalization. Of the five participants who received Sham first, all showed generalization in Phase 1 and none in Phase 2. Of the four participants who received tDCS first, all generalized in Phase 1, but two also generalized in Phase 2 (when they received Sham). This could either mean that Sham increased generalization in both phases, or that administering tDCS in the first phase extended the generalization potential to the subsequent Sham phase. This latter possibility receives some support from group data, through the observation of larger item-specific improvement and generalization in the tDCS phase. Nevertheless, we reiterate that the results regarding tDCS are not conclusive, as it is not possible to distinguish between a real tDCS-induced modulation and a ceiling effect in the Sham condition. Furthermore, it should be highlighted that we report data from a relatively small sample. Considering the fact that response to tDCS is characterized by a large inter-subject variability (Horvath et al., 2014), replication with a larger sample is essential to support the findings reported in the current study.

## Conclusion

The ACTION protocol improved lexical retrieval for both treated and untreated verbs. With generalization considered as the ultimate goal of aphasia therapy (Dickey and Yoo, 2013), results highlight the importance of engaging explicit morphosyntactic knowledge during rehabilitation of verb retrieval. Item-specific improvement was considerably larger than improvement of untreated items, but all participants improved significantly on both sets of verbs. Improvement was more marked in the first phase of treatment. Even though this study was not designed to assess the timing constraints of therapy, results stress the need to investigate the time-course of both item-specific and generalized improvement. The effects of bi-cephalic tDCS administered concurrently with ACTION are to be interpreted carefully, but while a ceiling effect cannot be excluded, larger therapy effects were observed during tDCS than Sham, for treated and untreated verbs.

### Conflict of interest statement

The authors declare that the research was conducted in the absence of any commercial or financial relationships that could be construed as a potential conflict of interest.

## Footnotes

^1^Given that electrodes of the same size were used over left and right hemisphere areas, the studies of Marangolo et al. (2013a, 2014) provide evidence for the efficacy of a bi-cephalic montage with anode over peri-lesional and cathode over contro-lesional areas. For a more detailed discussion see de Aguiar et al. (2015).

^2^Handedness was reported in the neurological assessment of each patient, and confirmed by patient and/or caregiver.

^3^Patients PG and GD were tested in consecutive days because they had to travel to participate in the study.

^4^Please note that the baseline phase is the same as the item selection phase. Had patients been tested only on difficult items, data would have been susceptible to the problem of regression to the mean. In our case, 88 verbs were tested in all assessments, even though at each treatment stage only 40 (20 treated and 20 untreated) were selected for statistical analyses. This approach allows an economic use of time, as only one pre-treatment phase is needed, and circumvents the problem of regression to the mean, making it possible to reliably assess both item-specific improvement and generalization (Howard et al., 2015).

^5^Whenever possible, only verbs that the patient failed to name but comprehended correctly were included in the treatment and control sets (see Supplementary Materials for exact numbers). Exceptions were made only when fewer than 40 such verbs were available to prepare the two sets, due to poor comprehension.

^6^This was the stimulation target for 8/9 patients.
