# Supplementary material for: Can tDCS enhance item-specific effects and generalization after linguistically motivated aphasia therapy for verbs?
Source: Front Behav Neurosci. 2015 Jul 30;9:190. doi: 10.3389/fnbeh.2015.00190 (PMC4519773; doi:10.3389/fnbeh.2015.00190)
Supplement: Supplementary file 1 [file DataSheet1.PDF]

*Supplementary Material*

# Item-specific and generalized effects of linguistically motivated aphasia therapy for verbs and tDCS

**Vânia de Aguiar[1,2,3], Roelien Bastiaanse[3], Rita Capasso[4], Marialuisa Gandolfi[5,6], Nicola Smania[5,6], Giorgio Rossi[7] and Gabriele Miceli[2]***

[1]International Doctorate in Experimental Approaches to Language And Brain (IDEALAB, Universities of Trento, Groningen, Potsdam, Newcastle and Macquarie University),
[2]Center for Mind/Brain Sciences (CIMeC) and Center for Neurocognitive Rehabilitation (CeRIN), University of Trento, Rovereto, Italy,
[3]Center for Language and Cognition Groningen (CLCG), University of Groningen, Groningen, The Netherlands,
[4]S.C.A. Associates, Rome.
[5]Neuromotor and Cognitive Rehabilitation Research Centre (CRRNC), USO Neurological Rehabilitation, AOUI of Verona, Verona, Italy,
[6]Department of Neurological and Movement Sciences, University of Verona, Verona, Italy
[7]Neurology, Santa Maria del Carmine Hospital, Rovereto, Italy

*** Correspondence:** Gabriele Miceli, CiMeC (Center for Mind/Brain Sciences), University of Trento, Palazzo Fedrigotti - corso Bettini 31, 38068 Rovereto (TN).
gabriele.miceli@unitn.it

## 1. Lesion descriptions

### 1.1. LF

Partial involvement of the inferior frontal gyrus, extending to deep structures, including the head of the caudate; extensive temporal damage, involving the pole, the superior, middle and (partly) inferior temporal gyrus and the temporo-occipital junction, and extending to the insula, claustrum, external capsule; massive involvement of angular and supramarginal gyrus, superior and inferior parietal lobule. The left lateral ventricle is markedly dilated.

### 1.2. GC

Angular and supramarginal gyrus; planum polare and planum temporale extending into the insula; middle temporal gyrus extending to the temporo-occipital junction; postcentral gyrus. Damage involves cortical structures and, extensively, the underlying white matter, with the exception of the insula, where damage is more superficial. The anterior portions of the superior and middle temporal gyrus are partially spared; damage to the superior aspect of the superior temporal gyrus and to the angular and supramarginal gyri spares cortical tissue and mostly affects subcortical structures.

### 1.3. GD

Sequelae of a vast intraparenchimal, left temporal hemorrage (anteroposterior diameter: approximately 8 cm). Damage involves the temporal lobe and the temporoparietal junction (temporal pole, superior and middle temporal gyrus, extending to the angular and supramarginal gyrus), and is associated with marked dilation of the temporal horn of the lateral ventricle). Additional (probably post-traumatic), mild right-hemisphere damage to basal and medial frontal areas, to mesial parietal areas and to the anterolateral portions of the temporal lobe. DTI shows damage to the white matter of the left hemisphere, interrupting the arcuate fasciculus almost entirely and damaging the inferior fronto-occipital fasciculus, the inferior longitudinal fasciculus and the uncinate (only a minimal number of streamlines of the latter can be recovered). All these fiber bundles are fully reconstructed in the right hemisphere.

### 1.4. GP

Extensive damage to the anterior branches of the left middle cerebral artery. The lesion massively affects frontal and temporal regions. In the temporal lobe, the pole is entirely disrupted, and damage affects the superior, middle and inferior temporal gyri, to a decreasing extent (the lesion destroys the entire superior temporal gyrus, but only the anterior half of the inferior temporal gyrus). In the frontal lobe, damage disrupts entirely the inferior and middle gyri, but affects the superior gyrus only marginally. Frontal and temporal damage affects all the white and grey matter structures underlying the affected cortex, all the way to the ventricular ependyma. Damage partially extends to the angular and supramargimal gyri.

### 1.5. EC

Sequelae of a hemorrhage seated deeply in the left hemisphere, centered around the lenticular nucleus (head of the caudate, putamen, pallidus, anterior portion of the thalamus), and extending superiorly to the level of the roof of the lateral ventricle. The post-hemorrage cavity is surrounded by white matter damage. Damage affects most of the insula, a sizeable portion of the inferior frontal gyrus (especially subcortically) and part of the planum temporale. Subcortically, the lesion extensively disrupts critical fiber tracts (direct and indirect segments of the arcuate fasciculus, inferior longitudinal fasciculus, inferior fronto-occipital fasciculus, uncinate fasciculus, corona radiata). Very marked *ex-vacuo* dilation of the lateral ventricle is present.

### 1.6. SP

Massive damage to the entire middle cerebral artery territory. The lesion involves the inferior and middle frontal gyrus, the insula, the inferior and superior parietal lobule, the superior and middle temporal gyrus (sparing the temporal pole), the angular and supramarginal gyrus, remarkably sparing the motor cortex and the anterior aspect of the post-central gyrus, and the corona radiata. Damage involves both the cortex and the subcortical white matter.

### 1.7. RL

Extensive lesion in the territory of the anterior branches of the left middle cerebral artery. Damage involves the inferior frontal gyrus (pars opercularis and pars triangularis), the middle

frontal gyrus, precentral and postcentral cortices, the superior temporal gyrus and the head of the hippocampus. It extends to the insula and to deep grey matter nuclei (caudate, globus pallidus, thalamus), also involving white matter tracts. Mild dilation of the left lateral ventricle is present.

### 1.8. CK

Damage followed a basal ganglia hemorrage and is almost entirely subcortical. The post-hemorrage cavity centers around the basal ganglia (head of the caudate, putamen, pallidus, internal capsule). It extends superiorly to the level of the roof of the lateral ventricle, and is surrounded by a large gliotic area. The site of the hemorrage is such that in all likelihood it undercuts most critical white matter bundles (arcuate fasciculus, inferior longitudinal fasciculus, corona radiata/internal capsule, possibly the uncinate and part of the inferior fronto-occipital fasciculus). Cortical damage is limited to the insula.

### 1.9. PG

Massive lesion in the territory of the parietotemporal branches of the middle cerebral artery. Damage spares almost entirely the prerolandic regions above the sylvian fissure, but extensively affects the temporal lobe (pole, superior temporal gyrus, middle temporal gyrus and the anterior half of the inferior temporal gyrus), the temporoparietal junction (angular and supramarginal gyrus), the parietal lobe (postcentral gyrus, superior and inferior parietal lobule) and the temporo-occipital junction. Temporal damage spares the middle portion of the pole, the hippocampus, the lingual and fusiform gyrus. The temporal isthmus and the insula are marginally involved; the temporal and occipital horn of the lateral ventricle are moderately dilated.

## 2. Diagnostic assessments

### 2.1. LF

LF presents non-fluent speech characterized by slow, laborious and often imprecise articulation. His output consists mostly of isolated noun phrases, with frequent pauses, word fragments, phonemic and semantic paraphasias. The informative value of his production is overall low, even though he uses nonverbal strategies to increase communicative efficacy. He performs below norm in all diagnostic tasks. Auditory discrimination is mildly impaired. Mild-to-moderate difficulty in all sublexical tasks and a mild length effect suggest damage to all sublexical conversion mechanisms. Mild impairment is observed for auditory and visual lexical decision, as well as for verb and noun comprehension. Oral and written naming of both nouns and verbs is more severely impaired than input tasks. Naming errors result mostly in anomias, as well as in phonemic and semantic paraphasias. Given the substantially greater impairment in naming (66.7% errors for nouns and 71.4% for verbs) than in comprehension tasks (10% errors for nouns and verbs), at least some naming errors are more likely to arise at a post-semantic stage (either at the level of access to the lexicons from semantic, or at the output lexicon stage). Segmental errors in all spoken output tasks suggest that the phonological working memory may also be

compromised. Oral naming of nouns and verbs is impaired to a similar degree (Fisher exact p=1.000). At the sentence level, morphological errors and errors of thematic role assignment are observed in comprehension (7/9 errors) and production. Sentence construction may be disrupted due to a complex deficit - reduced working memory, sublexical processing deficits (phonemic paraphasias), difficulties of lexical retrieval, and of grammatical encoding (thematic role reversals, argument omissions, morphosyntactic errors (e.g., determiner-noun agreement).

### 2.2. GC

GC presents fluent, effortless, well-articulated speech with appropriate speed and prosodic contour. Length of utterances is normal and informative content is adequate, but occasional phonemic and semantic paraphasias as well as word fragments and circumlocutions are observed. Auditory discrimination is mildly impaired. Though no length effect is observed, non-word repetition, reading and writing are below norm, consistent with damage to sublexical conversion mechanisms. Auditory and visual lexical decision are mildly impaired, but auditory and visual word comprehension are within norm, suggesting substantially unimpaired semantic processing. Pathological performance in writing to dictation, written object naming and word copying is consistent with an impairment of post-lexical and more peripheral processes (orthographic working memory, or later, writing-specific processes). Naming is impaired for verbs and nouns to a similar extent (20% errors for objects and 28.6% errors for actions; Fisher exact p=0.682). Errors consist of anomias, semantic paraphasias, visual and unrelated-word errors. Considering normal performance in word comprehension tasks (verbs: 0.0% errors; nouns: 2.5% errors), naming difficulties for nouns and verbs are very likely to arise at lexical, post-semantic levels. Auditory sentence comprehension is mildly impaired, with one error of thematic role assignment, one error on morphological foils and two errors on semantic foils. In sentence construction, difficulty with passives is observed, resulting in omissions of the auxiliary and thematic role reversals. There are also conduites d'approche, morphologically related words, circumlocutions, semantic and phonemic paraphasias. In the light of associated deficits in sentence repetition, results suggest that sentence production difficulties result from a complex impairment affecting sublexical, lexical and grammatical encoding, as well as working memory.

### 2.3. GD

GD presents fluent, effortless speech with appropriate articulation, prosody and speed. Sentences are of adequate length, but frequent semantic paraphasias and circumlocutions reduce their informative value. Auditory discrimination is below norm. Nonword repetition is relatively more impaired, consistent with damage to phoneme/phoneme conversion mechanisms. Auditory lexical decision and auditory noun comprehension (20% errors) are both mildly impaired. Auditory comprehension of verbs and visual comprehension of nouns and verbs are normal. In a picture verification task (described in the Methods section), GD makes errors on semantic foils, suggesting mild semantic impairment. Comparably severe naming difficulty for nouns and verbs (60% errors for nouns and 57.1% for verbs; Fisher exact p=1.000) may then arise from a semantic, or post-semantic deficit involving the phonemic output lexicon. Impairment in all spoken output tasks (naming, reading aloud, word and non-word repetition) and a length effect in non-word repetition, are consistent with an impairment of phonological working memory.

Accordingly, errors in sentence repetition occur mostly at the end of the sentence. Sentence comprehension is mildly impaired, with three errors of thematic role inversion. Thematic role reversals are also observed in sentence production, together with omission of the auxiliary and by-phrase in passive constructions. Both the lexical verb and its argument are frequently omitted, and semantic paraphasias occur.

### 2.4. GP

GP presents non-fluent, slow, effortful speech, with appropriate prosody and precise articulation. He produces very short sentences, mostly consisting of isolated noun phrases. He produces very few verbs, in non-finite forms. Nevertheless, he is able to convey complex messages (e.g., plans for the coming holidays) using telegraphic sentences. He performs below norm in all sublexical processing tasks (except non-word copying), suggesting impairment to phoneme/phoneme, phoneme/grapheme, and grapheme/phoneme conversion mechanisms. Performance is below norm in auditory and visual lexical decision, but comprehension is only impaired in the visual modality, for verbs. This suggests normal or mildly impaired semantic processing. Spoken and written naming are impaired for nouns and verbs. Oral naming impairment is significantly more severe for verbs (16.7% errors for nouns and 78.6% for verbs; Fisher exact $p<0.001$). Paired with intact comprehension, poor naming suggest that naming difficulties arise at a post-semantic locus (lexical access and/or storage). In addition, GP performs below norm in all oral output tasks, shows a length effect in naming and produces occasional phonemic paraphasias, consistent with additional damage to the phonemic output buffer. In sentence comprehension GP makes errors with thematic role assignment, and with morphological and semantic distractors. In sentence production he tends to reverse thematic roles. Most errors occur on the verb, and involve both verb retrieval (nominalizations, perseverations and, less often, omissions) and inflectional processes (subject-verb agreement errors, mostly resulting in the production of a non-finite verb form).

### 2.5. EC

EC presents non-fluent, slow, effortful speech, with reduced prosodic contour and accurate articulation. He produces short sentences, filled with pauses, repairs and omissions, with low informative value. He performs below norm in all sublexical processing tasks, except auditory discrimination. These scores reflect widespread impairment of sublexical conversion mechanisms. Auditory and visual lexical decision is impaired, but auditory and visual comprehension of nouns and verbs is intact, suggesting spared semantic processing. Below norm performance in word writing (both to dictation and in written naming) and word copy suggests damage to peripheral processes (graphemic output buffer, allographic or graphomotor realization). Oral object and action naming are similarly impaired (43.3% errors for nouns and 57.1% for verbs; Fisher exact $p=0.431$), resulting in anomias, and (less frequently) in semantic and phonemic paraphasias. The naming difficulty seems to arise from damage to lexical mechanisms. In sentence comprehension, EC makes two errors of thematic role reversal, and incorrectly selects two morphological and two semantic foils. In sentence production, EC omits

arguments, and in passive sentences replaces the past participle construction with a reflexive construction. He also produces occasional semantic paraphasias.

### 2.6. SP

SP presents non fluent speech, characterized by slow and imprecise articulation, and short sentences with preserved prosodic contour. Paraphasias occur frequently and consist most often of words unrelated to the target, semantic paraphasias or, less often, neologistic and phonemic paraphasias. Stereotypical phrases are present and so is palilalia. His output conveys very little information, and he is often unaware of his errors. Due to difficulty in task comprehension, several diagnostic tasks were not administered. Phoneme/phoneme and grapheme/phoneme conversion are impaired, as shown by poor scores in sublexical processing tasks. Auditory and visual lexical decision are below norm, denoting input lexicon damage. Modality-independent difficulties in input and output word processing tasks suggest semantic damage. Action and object naming are impaired to a similar extent (60% errors for nouns and 64.3% for verbs; Fisher exact p=1.000), and word frequency affects performance. Naming difficulties in this subject may arise at semantic and/or post-semantic levels (access to or processing within the phonological output lexicon). Scores are below norm in all output tasks and there is a mild length effect, suggesting additional post-lexical impairment. Sentence comprehension is severely impaired, with errors occurring in stimuli presented with semantic (n=3), morphological (n=5) and role reversal foils (n=4). In sentence production, SP produces mostly nouns, both related and unrelated to targets. When verbs are produced, they are often unrelated to the target and inappropriate to its predicate argument structure, consistent with a semantic deficit. Function words and verbs are frequently omitted.

### 2.7. RL

RL's speech is non-fluent, slow and effortful, with a mild articulation deficit, and normal prosody. She produces short sentences with frequent pauses, semantic paraphasias and word fragments. Nevertheless, she makes good use of verbal and non-verbal strategies and communication is usually sufficiently informative. All sublexical processing tasks except for nonword copying are mildly-to-moderately impaired, as a consequence of damage to phoneme/phoneme, phoneme/grapheme and grapheme/phoneme conversion. Auditory lexical decision is only mildly impaired, and auditory and visual word comprehension is within norm, suggesting spared semantic processes. Written naming is particularly impaired, but even though spoken naming is within norm, correct performance is achieved after multiple naming attempts and only if time-to-respond is not taken into account. We considered first-response accuracy in assessing verb production performance, and RL produced 37.5% errors when retrieving verbs in the infinitive in sentence context. Most errors on first attempt are word fragments, semantic and phonemic paraphasias and occasionally, nominalizations or substitutions of a non-related verb for the target. A post-semantic, lexical deficit is likely in this subject. In addition, mild impairment in word and sentence repetition, and the length effect in non-word repetition are consistent with a mild disorder of phonological working memory. Sentence comprehension (auditory and visual) is normal. Sentence production was assessed with VTsentence. Responses were scored for first-response accuracy, with a time limit of 30 seconds per sentence. The most

frequent errors were of the semantic and anomic types, followed by word fragments and phonemic paraphasias. Attempts were generally made to produce each constituent (11.6% of omissions), revealing good knowledge of verb argument structure. Sentence production seems to be impaired mostly due to lexical retrieval and post-lexical impairments.

## 2.8. KC

KC presents non-fluent, slow, effortful, dysarthric speech with very low informative value. Prosody is adequate. Communicative initiative is also low. Auditory discrimination is mildly impaired. Nonword repetition, reading and writing are below norm, suggesting damage to phoneme/phoneme, grapheme/phoneme and phoneme/grapheme conversion. Scores below norm in auditory lexical decision, associated with a frequency effect, indicate that poor auditory comprehension can partially be accounted for by deficits in the phonological input lexicon. In addition, poor performance in all (input and output) tasks that require access to word meanings is consistent with damage to the semantic system. Oral naming is comparably impaired for nouns and verbs (13.3% errors for nouns and 14.3% for verbs; Fisher exact p=1.000). In sentence comprehension, KC makes errors with semantic and thematic role distractors. In sentence production, she tends to use light verbs or to omit the lexical verb and one or more of its arguments. Her speech contains word fragments, semantic, phonemic and neologistic paraphasias, and occasionally, unrelated words. In addition, if forced to use passives, she produces thematic role assignment errors. Sentence repetition is also impaired, and errors in repetition tasks are influenced by word length, suggesting reduced phonological working memory.

## 2.9. PG

PG's speech is fluent, effortless, with adequate articulation, prosody, speed, and sentence length. He conveys information appropriately, although he resorts to repeated attempts at production, including circumlocutions and reformulations. Sublexical phoneme/phoneme, grapheme/phoneme, and phoneme/grapheme conversion are impaired. While scores in auditory and visual lexical decision are slightly below norm, phoneme discrimination is within norm. This suggests mild damage to the phonological and orthographic input lexicons. Auditory and visual comprehension of nouns and verbs are within norm, consistent with spared semantic processing. Oral and written naming is impaired for nouns and verbs (to a similar extent, 33.3% errors for nouns and 42.9% for verbs; Fisher exact p=0.710), suggesting post-semantic damage. Pathological word reading is consistent with damage to the phonological output lexicon. The high proportion of segmental errors in delayed word copy is consistent with post-lexical damage. Impaired sentence repetition and effects of length in sublexical conversion tasks suggest reduced working memory. In sentence comprehension, most errors result from thematic role reversals, but also occur when target sentences are presented with morphological foils. In sentence production, fragments, and circumlocutions are frequent. In addition, PG produces frequent phonemic paraphasias, followed by conduites d'approche.

### 3. Cueing procedure use in ACTION steps 3 and 4

In Step 3, the participant saw an image with an adverb and a subject written below the picture (e.g., "Now the man…"), and was asked to complete the sentence with the verb inflected in the correct tense. If the subject failed to retrieve the correct verb, increasing cues were provided depending on error type, following a structured schema (Supplementary Figure 1).

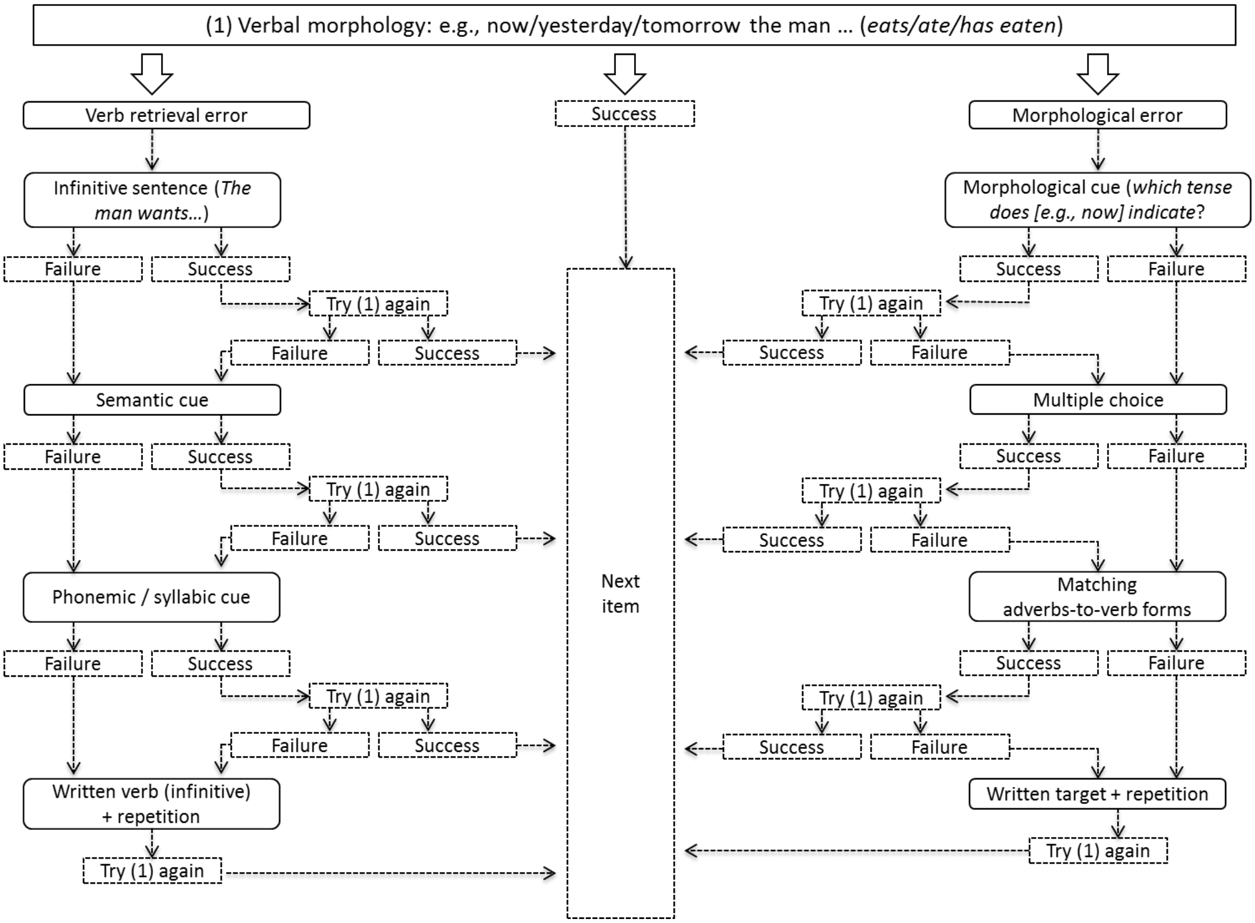


**Supplementary Figure 1. Cueing hierarchy for Step 3**. Cues for verb retrieval and for the production of verb morphology were provided depending on error type.

a) The participant was presented with a sentence to be completed with an infinitive verb ("The man wants…"). If the correct verb was retrieved, Step 3 was tried again. In the event of a successful attempt, to the next item was presented. If case of failure, the therapist proceeded to (b).

b) The participant was presented with a semantic cue, related to the function or characteristics of the action. The semantic cue was a semantically loaded sentence that led to produce the infinitive. If retrieval was successful, the participant tried Step 3 again and, in case of correct response, the therapist went on to the next item. In the case of failure, the therapist proceeded to (c).

c) A phonemic cue (initial sound) was added to the semantic cue. If it did not precipitate the correct response, the whole first syllable was produced by the therapist (syllabic cue). If the correct verb was retrieved, the participant tried Step 3 again. If the attempt was successful, the therapist went on to the next item. If the participant failed the therapist proceeded to (d).

d) A card with the written verb in the infinitive was provided and at the same time the therapist said the word aloud. The participant was asked to repeat/read the target verb in the infinitive. The cue was presented until the participant succeeded to read/repeat (in case of excessive frustration, the therapist moved on to the following verb). After producing the verb the participant tried Step 3 again. In case of success the therapist moved to the next item; in case of failure (d) was provided again. Then the therapist administered the following item, even if the response was not correct.

In Step 4, the participant saw an image and a written adverb (e.g., "now…"), and was requested to produce a full sentence that properly described the image (SVO or SOA), with the verb in the correct tense. If no response was provided, the following cues were used (Supplementary Figure 2):

a) The participant was asked to name each constituent, prompted by a question: for the subject "Who does this action?"; for the verb "What is the action/the verb?"; for the object "What is the object/the thing?" or adjunct "Where does this happen?". The therapist started by asking the participant to name the constituents that had been retrieved successfully. Those to which the subject had failed to produce any response were the last to be prompted. If no constituent was named correctly, subject, object/adjunct and then verb were presented, in this order. If the participant succeeded in naming each word, Step 4 was tried again. If retrieval errors prevailed, verb retrieval cueing proceeded with (b) and cueing of the subject or object/theme with (c).

b) The participant was presented with a sentence to be completed with an infinitive verb ("The man wants…"). If the participant failed to retrieve the verb, the therapist proceeded to (c). In case of success, the remaining constituents were named and then Step 4 was repeated. If retrieval errors persisted, cue (c) was provided. In case of success, the therapist proceeded to the following item.

c) The participant was presented with a semantic cue, that is, a semantically-loaded sentence (with information about the function or other features of the target word) that led to producing the target word (for the verb, in the infinitive). If the participant still failed to retrieve the word, the therapist proceeded to (d). Upon success, the participant named the other constituents and tried Step 4 again. If no errors occurred, the therapist proceeded to the next item. If the participant still failed, the therapist proceeded to (d).

d) A phonemic cue was added to the semantic cue. If this did not help, the whole first syllable was produced by the therapist (syllabic cue). If the participant failed, (e) was provided. If the correct word was retrieved, the participant named the other constituents

and then tried Step 4 again. If no errors occurred the therapist went on to the next item. If the participant failed, the therapist proceeded to (e).

e) A card with the written word (for the verb, the infinitive form) was provided and at the same time the therapist said the word aloud. The participant repeated/read the target word. The cue was presented until the participant succeeded to read/repeat (but if the participant was too frustrated, the therapist moved on to the next item). After producing the word the participant named the remaining constituents, and then tried Step 4 again. If no errors occurred the therapist went on to the next item. In case of failure, the therapist proceeded to (f).

f) Sentence anagrams: the participant saw 3 cards with the 3 sentence constituents, in random order. The participant arranged the constituents to form the correct sentence, and then read it aloud. If the participant failed, the therapist ordered the constituents correctly and asked the participant to read the sentence aloud. Subsequently, the three cards were removed and the participant tried Step 4 again. After this attempt, the therapist moved on to the next item, even if the response was not correct.

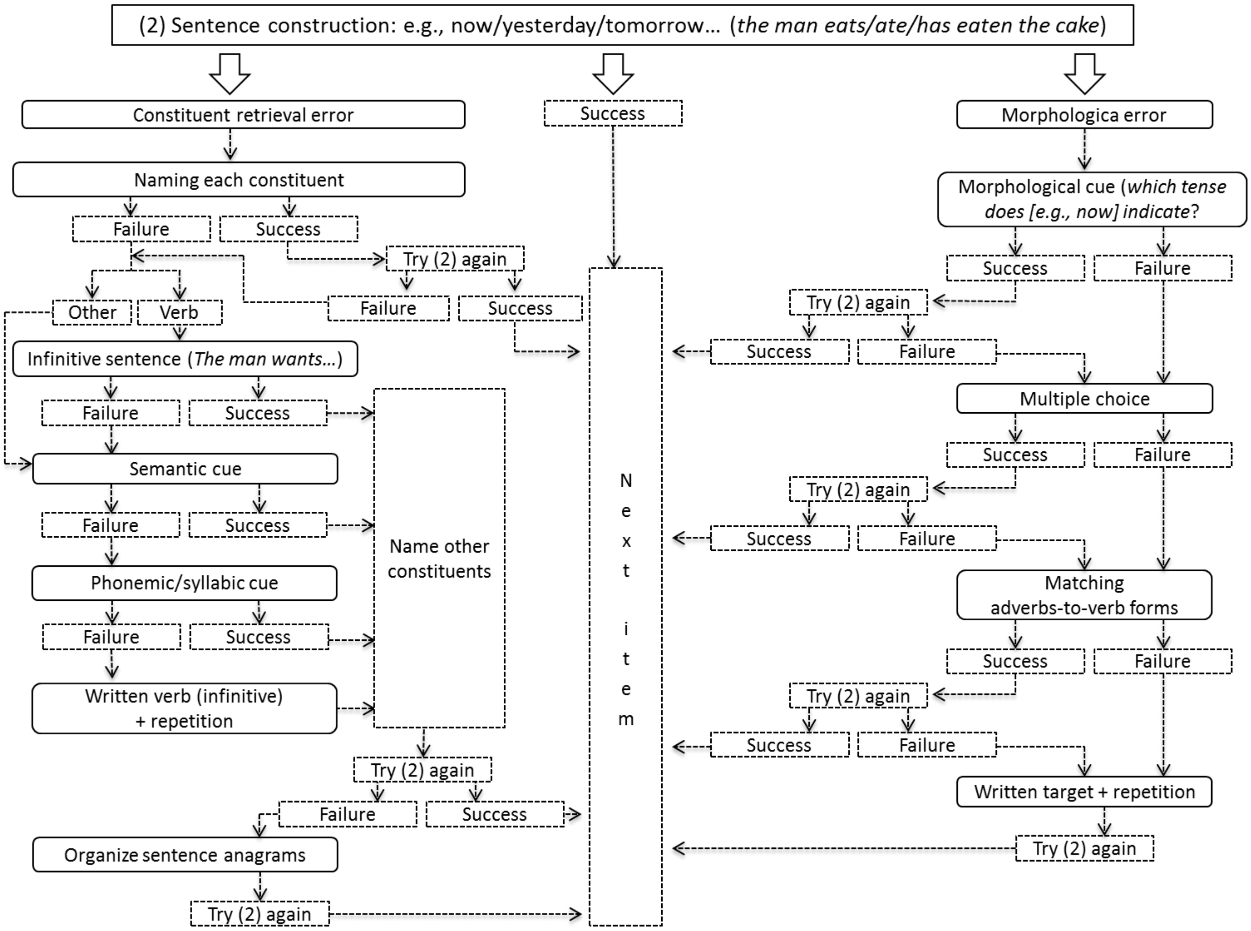


**Supplementary Figure 2. Cueing hierarchy for Step 4**. Cues for verb retrieval and for the production of verb morphology were provided depending on error type.

When participants produced morphological errors, the following cues were given in both Steps 3 and 4:

a) The participant was asked the following question “which tense does X indicate?”, where X is the adverb that was provided. The participant could reply verbally, or indicate the correct option on a sheet of paper, as long as knowledge of the correct time reference could be verified. If the correct tense was indicated, the participant tried again. If the participant succeeded, the therapist moved on to the next item; if the morphological error persisted, the therapist proceeded to (b). If the wrong tense was indicated, the therapist provided the correct information (e.g. “Now indicates the present tense”) and moved on to (b).

b) Multiple-choice: the therapist provided three cards with three verb forms. The participant was asked to choose the card was correct for the presented adverb. After selecting the correct option the participant read it aloud. The card was then hidden and the participant tried step 3 or 4 again. If the response was correct, the therapist moved on to the next item; if the morphological error still occurred, the therapist moved on to (c). When the participant chose the wrong tense card the therapist moved on to (c).

c) Adverb/verb-form matching: the therapist placed the card for each adverb on the table while saying the time-frame indicated by the adverb (e.g., “Now indicates the present”) and placed the cards for each verb form while also saying the time-frame that form indicated (e.g., “eats indicates the present”). Adverbs were placed in a column and verbs in another column, in mismatching positions, and the participant was asked to place each verb-form next to the corresponding adverb. If matching was correct, the participant tried Step 3 or 4 again and upon success the therapist moved on to the next item; if the morphological error still occurred, the therapist moved on to (d). If the participant failed to provide the correct matching, the therapist performed it and then moved on to (d).

d) The therapist provided cards with the adverb and the inflected verb and completed the sentence with the correctly inflected verb (“Now the man eats” or “Now the man eats the pie.”, for Steps 3 and 4, respectively). The participant repeated/read the correctly inflected verb. Then the participant was asked to try again. If the participant was successful, the therapist moved on to the next item; otherwise (d) was provided again.

**4. Item matching, for each treatment phase, for each participant (based on norms from Rofes, de Aguiar, & Miceli, *in press*)**

**Supplementary Table 1. Matching of treated and untreated verbs for psycholinguistic variables: LF**

| | Phase 1 | | | | | Phase 2 | | | | |
|---|---|---|---|---|---|---|---|---|---|---|
| | Untreated | | Treated | | T-test | Untreated | | Treated | | T-test |
| | *Mean* | *std* | *Mean* | *std* | *P-value* | *Mean* | *std* | *Mean* | *std* | *P-value* |
| Sentence agreement | 88.00% | 0.077 | 88.50% | 0.081 | 0.843 | 89.50% | 0.083 | 90.50% | 0.076 | 0.692 |
| Age of Acquisition | 2.184 | 0.604 | 2.079 | 0.473 | 0.546 | 2.100 | 0.475 | 1.977 | 0.730 | 0.533 |
| Imageability | 1.401 | 0.314 | 1.419 | 0.421 | 0.878 | 1.366 | 0.315 | 1.300 | 0.178 | 0.419 |
| Relative frequency | 30.809 | 47.520 | 27.395 | 33.734 | 0.795 | 53.186 | 85.751 | 59.012 | 80.649 | 0.826 |
| Length in phonemes | 7.900 | 1.252 | 8.250 | 1.372 | 0.405 | 8.100 | 1.252 | 8.150 | 1.268 | 0.901 |
| | *Count* | | *Count* | | | *Count* | | *Count* | | |
| Transitivity | 14 | | 14 | | | 13 | | 13 | | |
| Internal arguments | 14 | | 14 | | | 13 | | 13 | | |
| Instrumental | 12 | | 10 | | | 8 | | 9 | | |
| Name related | 4 | | 3 | | | 3 | | 2 | | |
| Manipulable | 14 | | 12 | | | 11 | | 11 | | |
| *Face/arm/leg* | | | | | | | | | | |
| *face actions | 2 | | 1 | | | 1 | | 3 | | |
| *arm actions | 14 | | 16 | | | 14 | | 13 | | |
| *leg actions | 0 | | 1 | | | 2 | | 3 | | |
| *face&arm actions | 1 | | 0 | | | 0 | | 0 | | |
| *arm&leg actions | 0 | | 0 | | | 1 | | 0 | | |
| *"NA" actions | 3 | | 2 | | | 2 | | 1 | | |
| *Conjugation* | | | | | | | | | | |
| *first | 16 | | 16 | | | 15 | | 14 | | |
| *second | 2 | | 1 | | | 3 | | 4 | | |
| *third | 2 | | 3 | | | 2 | | 2 | | |

**Supplementary Table 2. Matching of treated and untreated verbs for psycholinguistic variables: GC**

| | Phase 1 | | | | | Phase 2 | | | | |
|---|---|---|---|---|---|---|---|---|---|---|
| | Untreated | | Treated | | T-test | Untreated | | Treated | | T-test |
| | *Mean* | *std* | *Mean* | *std* | *P-value* | *Mean* | *std* | *Mean* | *std* | *P-value* |
| Sentence agreement | 92.00% | 0.083 | 88.50% | 0.081 | 0.187 | 90.00% | 0.086 | 89.00% | 0.079 | 0.703 |
| Age of Acquisition | 2.052 | 0.588 | 2.232 | 0.541 | 0.320 | 2.098 | 0.688 | 2.034 | 0.496 | 0.739 |
| Imageability | 1.425 | 0.323 | 1.352 | 0.327 | 0.483 | 1.379 | 0.351 | 1.331 | 0.127 | 0.567 |
| Relative frequency | 25.391 | 31.264 | 24.940 | 36.034 | 0.966 | 23.406 | 24.878 | 21.564 | 32.208 | 0.841 |
| Length in phonemes | 8.200 | 1.056 | 8.050 | 1.432 | 0.708 | 8.550 | 1.356 | 7.850 | 1.137 | 0.085 |
| | *Count* | | *Count* | | | *Count* | | *Count* | | |
| Transitivity | 15 | | 14 | | | 14 | | 14 | | |
| Internal arguments | 15 | | 14 | | | 14 | | 14 | | |
| Instrumental | 10 | | 11 | | | 11 | | 10 | | |
| Name related | 3 | | 2 | | | 4 | | 4 | | |
| Manipulable | 12 | | 13 | | | 13 | | 13 | | |
| *Face/arm/leg* | | | | | | | | | | |
| *face actions | 1 | | 1 | | | 2 | | 1 | | |
| *arm actions | 15 | | 15 | | | 14 | | 14 | | |
| *leg actions | 0 | | 1 | | | 1 | | 0 | | |
| *face&arm actions | 0 | | 0 | | | 1 | | 1 | | |
| *arm&leg actions | 1 | | 0 | | | 0 | | 1 | | |
| *"NA" actions | 2 | | 2 | | | 2 | | 3 | | |
| *Conjugation* | | | | | | | | | | |
| *first | 16 | | 16 | | | 16 | | 16 | | |
| *second | 1 | | 2 | | | 2 | | 2 | | |
| *third | 3 | | 2 | | | 2 | | 2 | | |

**Supplementary Table 3. Matching of treated and untreated verbs for psycholinguistic variables: GD**

| | Phase 1 | | | | | Phase 2 | | | | |
|---|---|---|---|---|---|---|---|---|---|---|
| | Untreated | | Treated | | T-test | Untreated | | Treated | | T-test |
| | *Mean* | *std* | *Mean* | *std* | *P-value* | *Mean* | *std* | *Mean* | *std* | *P-value* |
| Sentence agreement | 91.50% | 0.075 | 89.00% | 0.085 | 0.330 | 92.00% | 0.077 | 91.50% | 0.093 | 0.854 |
| Age of Acquisition | 1.958 | 0.547 | 1.885 | 0.575 | 0.683 | 1.849 | 0.391 | 1.961 | 0.600 | 0.489 |
| Imageability | 1.292 | 0.159 | 1.344 | 0.433 | 0.613 | 1.354 | 0.333 | 1.387 | 0.324 | 0.758 |
| Relative frequency | 37.343 | 49.045 | 42.514 | 75.243 | 0.798 | 42.173 | 45.499 | 37.188 | 44.657 | 0.729 |
| Length in phonemes | 8.000 | 1.414 | 7.750 | 1.517 | 0.593 | 7.900 | 1.294 | 7.750 | 1.118 | 0.697 |
| | *Count* | | *Count* | | | *Count* | | *Count* | | |
| Transitivity | 13 | | 13 | | | 12 | | 12 | | |
| Internal arguments | 13 | | 13 | | | 12 | | 12 | | |
| Instrumental | 9 | | 8 | | | 9 | | 9 | | |
| Name related | 1 | | 3 | | | 3 | | 3 | | |
| Manipulable | 12 | | 11 | | | 11 | | 11 | | |
| *Face/arm/leg* | | | | | | | | | | |
| *face actions | 1 | | 1 | | | 2 | | 3 | | |
| *arm actions | 13 | | 11 | | | 11 | | 14 | | |
| *leg actions | 2 | | 2 | | | 3 | | 2 | | |
| *face&arm actions | 1 | | 1 | | | 1 | | 0 | | |
| *arm&leg actions | 1 | | 0 | | | 1 | | 0 | | |
| *"NA" actions | 2 | | 5 | | | 2 | | 1 | | |
| *Conjugation* | | | | | | | | | | |
| *first | 16 | | 15 | | | 15 | | 16 | | |
| *second | 3 | | 3 | | | 3 | | 3 | | |
| *third | 1 | | 2 | | | 2 | | 1 | | |

**Supplementary Table 4. Matching of treated and untreated verbs for psycholinguistic variables: GP**

| | Phase 1 | | | | | Phase 2 | | | | |
|---|---|---|---|---|---|---|---|---|---|---|
| | Untreated | | Treated | | T-test | Untreated | | Treated | | T-test |
| | *Mean* | *std* | *Mean* | *std* | *P-value* | *Mean* | *std* | *Mean* | *std* | *P-value* |
| Sentence agreement | 91.50% | 0.081 | 91.50% | 0.081 | 1.000 | 91.00% | 0.091 | 91.50% | 0.075 | 0.850 |
| Age of Acquisition | 2.067 | 0.598 | 2.036 | 0.594 | 0.868 | 1.953 | 0.605 | 1.910 | 0.493 | 0.807 |
| Imageability | 1.384 | 0.308 | 1.345 | 0.204 | 0.639 | 1.357 | 0.339 | 1.303 | 0.188 | 0.536 |
| Relative frequency | 28.643 | 36.505 | 28.880 | 36.208 | 0.984 | 40.351 | 47.126 | 46.047 | 54.615 | 0.726 |
| Length in phonemes | 7.550 | 1.432 | 7.550 | 1.050 | 1.000 | 7.750 | 1.517 | 7.600 | 1.095 | 0.722 |
| | *Count* | | *Count* | | | *Count* | | *Count* | | |
| Transitivity | 15 | | 15 | | | 14 | | 14 | | |
| Internal arguments | 15 | | 15 | | | 14 | | 14 | | |
| Instrumental | 8 | | 9 | | | 9 | | 11 | | |
| Name related | 3 | | 3 | | | 2 | | 3 | | |
| Manipulable | 13 | | 12 | | | 12 | | 12 | | |
| *Face/arm/leg* | | | | | | | | | | |
| *face actions | 1 | | 2 | | | 0 | | 3 | | |
| *arm actions | 12 | | 14 | | | 14 | | 13 | | |
| *leg actions | 2 | | 1 | | | 1 | | 3 | | |
| *face&arm actions | 2 | | 1 | | | 2 | | 0 | | |
| *arm&leg actions | 0 | | 0 | | | 0 | | 0 | | |
| *"NA" actions | 3 | | 2 | | | 2 | | 1 | | |
| *Conjugation* | | | | | | | | | | |
| *first | 16 | | 15 | | | 15 | | 15 | | |
| *second | 2 | | 2 | | | 3 | | 4 | | |
| *third | 2 | | 3 | | | 2 | | 1 | | |

**Supplementary Table 5. Matching of treated and untreated verbs for psycholinguistic variables: EC**

| | Phase 1 | | | | | Phase 2 | | | | |
|---|---|---|---|---|---|---|---|---|---|---|
| | Untreated | | Treated | | T-test | Untreated | | Treated | | T-test |
| | *Mean* | *std* | *Mean* | *std* | *P-value* | *Mean* | *std* | *Mean* | *std* | *P-value* |
| Sentence agreement | 88.50% | 0.075 | 88.00% | 0.070 | 0.828 | 89.50% | 0.089 | 90.50% | 0.083 | 0.714 |
| Age of Acquisition | 2.124 | 0.449 | 2.087 | 0.513 | 0.812 | 2.005 | 0.518 | 2.002 | 0.626 | 0.987 |
| Imageability | 1.302 | 0.159 | 1.312 | 0.198 | 0.859 | 1.428 | 0.405 | 1.297 | 0.331 | 0.269 |
| Relative frequency | 23.741 | 35.207 | 21.366 | 24.570 | 0.806 | 41.679 | 72.521 | 46.439 | 87.876 | 0.853 |
| Length in phonemes | 7.850 | 1.040 | 7.750 | 1.209 | 0.781 | 8.050 | 1.356 | 7.600 | 1.353 | 0.300 |
| | *Count* | | *Count* | | | *Count* | | *Count* | | |
| Transitivity | 15 | | 15 | | | 14 | | 14 | | |
| Internal arguments | 15 | | 15 | | | 14 | | 14 | | |
| Instrumental | 11 | | 10 | | | 9 | | 11 | | |
| Name related | 4 | | 3 | | | 2 | | 3 | | |
| Manipulable | 14 | | 14 | | | 14 | | 13 | | |
| *Face/arm/leg* | | | | | | | | | | |
| *face actions | 1 | | 0 | | | 3 | | 3 | | |
| *arm actions | 18 | | 16 | | | 13 | | 14 | | |
| *leg actions | 1 | | 2 | | | 0 | | 0 | | |
| *face&arm actions | 0 | | 0 | | | 1 | | 1 | | |
| *arm&leg actions | 0 | | 1 | | | 0 | | 0 | | |
| *"NA" actions | 2 | | 1 | | | 4 | | 1 | | |
| *Conjugation* | | | | | | | | | | |
| *first | 16 | | 16 | | | 14 | | 14 | | |
| *second | 2 | | 2 | | | 4 | | 3 | | |
| *third | 2 | | 2 | | | 2 | | 3 | | |

**Supplementary Table 6. Matching of treated and untreated verbs for psycholinguistic variables: SP**

| | Phase 1 | | | | | Phase 2 | | | | |
|---|---|---|---|---|---|---|---|---|---|---|
| | Untreated | | Treated | | T-test | Untreated | | Treated | | T-test |
| | *Mean* | *std* | *Mean* | *std* | *P-value* | *Mean* | *std* | *Mean* | *std* | *P-value* |
| Sentence agreement | 89.50% | 0.089 | 92.50% | 0.079 | 0.265 | 90.00% | 0.086 | 92.00% | 0.077 | 0.442 |
| Age of Acquisition | 1.930 | 0.608 | 2.009 | 0.462 | 0.648 | 1.916 | 0.519 | 2.071 | 0.568 | 0.375 |
| Imageability | 1.333 | 0.196 | 1.271 | 0.143 | 0.257 | 1.381 | 0.414 | 1.359 | 0.188 | 0.825 |
| Relative frequency | 33.599 | 44.969 | 34.283 | 48.647 | 0.963 | 30.458 | 34.667 | 35.385 | 47.477 | 0.710 |
| Length in phonemes | 8.200 | 1.399 | 8.050 | 1.432 | 0.739 | 7.850 | 1.268 | 7.900 | 1.334 | 0.904 |
| | *Count* | | *Count* | | | *Count* | | *Count* | | |
| Transitivity | 13 | | 13 | | | 14 | | 15 | | |
| Internal arguments | 13 | | 13 | | | 14 | | 15 | | |
| Instrumental | 9 | | 7 | | | 10 | | 9 | | |
| Name related | 3 | | 2 | | | 2 | | 3 | | |
| Manipulable | 13 | | 8 | | | 13 | | 11 | | |
| *Face/arm/leg* | | | | | | | | | | |
| *face actions | 1 | | 3 | | | 1 | | 1 | | |
| *arm actions | 12 | | 10 | | | 13 | | 13 | | |
| *leg actions | 1 | | 3 | | | 2 | | 1 | | |
| *face&arm actions | 1 | | 1 | | | 0 | | 1 | | |
| *arm&leg actions | 1 | | 0 | | | 0 | | 1 | | |
| *"NA" actions | 3 | | 3 | | | 2 | | 3 | | |
| *Conjugation* | | | | | | | | | | |
| *first | 15 | | 16 | | | 15 | | 15 | | |
| *second | 3 | | 2 | | | 3 | | 3 | | |
| *third | 2 | | 2 | | | 2 | | 2 | | |

**Supplementary Table 7. Matching of treated and untreated verbs for psycholinguistic variables: RL**

| | Phase 1 | | | | | Phase 2 | | | | |
|---|---|---|---|---|---|---|---|---|---|---|
| | Untreated | | Treated | | T-test | Untreated | | Treated | | T-test |
| | *Mean* | *std* | *Mean* | *std* | *P-value* | *Mean* | *std* | *Mean* | *std* | *P-value* |
| Sentence agreement | 89.50% | 0.083 | 91.00% | 0.085 | 0.575 | 90.50% | 0.076 | 90.50% | 0.083 | 1.000 |
| Age of Acquisition | 2.006 | 0.568 | 1.968 | 0.623 | 0.843 | 2.080 | 0.526 | 2.071 | 0.440 | 0.956 |
| Imageability | 1.399 | 0.212 | 1.357 | 0.316 | 0.632 | 1.351 | 0.187 | 1.333 | 0.190 | 0.761 |
| Relative frequency | 44.113 | 75.507 | 38.218 | 43.068 | 0.763 | 38.108 | 75.190 | 26.581 | 39.205 | 0.547 |
| Length in phonemes | 7.800 | 1.196 | 7.650 | 1.182 | 0.692 | 7.800 | 1.576 | 8.000 | 1.076 | 0.642 |
| | *Count* | | *Count* | | | *Count* | | *Count* | | |
| Transitivity | 14 | | 14 | | | 14 | | 14 | | |
| Internal arguments | 14 | | 14 | | | 14 | | 14 | | |
| Instrumental | 9 | | 8 | | | 10 | | 12 | | |
| Name related | 2 | | 2 | | | 4 | | 4 | | |
| Manipulable | 10 | | 10 | | | 12 | | 12 | | |
| *Face/arm/leg* | | | | | | | | | | |
| *face actions | 3 | | 3 | | | 2 | | 3 | | |
| *arm actions | 13 | | 13 | | | 13 | | 13 | | |
| *leg actions | 0 | | 0 | | | 2 | | 2 | | |
| *face&arm actions | 0 | | 0 | | | 0 | | 0 | | |
| *arm&leg actions | 1 | | 0 | | | 0 | | 0 | | |
| *"NA" actions | 3 | | 4 | | | 2 | | 2 | | |
| *Conjugation* | | | | | | | | | | |
| *first | 15 | | 16 | | | 15 | | 14 | | |
| *second | 2 | | 2 | | | 2 | | 3 | | |
| *third | 3 | | 2 | | | 3 | | 3 | | |

**Supplementary Table 8. Matching of treated and untreated verbs for psycholinguistic variables: KC**

| | Phase 1 | | | | | Phase 2 | | | | |
|---|---|---|---|---|---|---|---|---|---|---|
| | Untreated | | Treated | | T-test | Untreated | | Treated | | T-test |
| | *Mean* | *std* | *Mean* | *std* | *P-value* | *Mean* | *std* | *Mean* | *std* | *P-value* |
| Sentence agreement | 88.00% | 0.077 | 89.50% | 0.083 | 0.555 | 90.50% | 0.083 | 91.00% | 0.085 | 0.852 |
| Age of Acquisition | 1.962 | 0.516 | 1.925 | 0.504 | 0.818 | 1.906 | 0.614 | 1.897 | 0.404 | 0.957 |
| Imageability | 1.386 | 0.423 | 1.362 | 0.342 | 0.846 | 1.401 | 0.484 | 1.349 | 0.221 | 0.664 |
| Relative frequency | 33.098 | 32.132 | 39.749 | 47.896 | 0.609 | 43.678 | 68.340 | 49.073 | 56.607 | 0.787 |
| Length in phonemes | 7.900 | 1.165 | 8.150 | 1.461 | 0.553 | 7.800 | 1.473 | 7.750 | 1.070 | 0.903 |
| | *Count* | | *Count* | | | *Count* | | *Count* | | |
| Transitivity | 12 | | 13 | | | 15 | | 15 | | |
| Internal arguments | 12 | | 13 | | | 15 | | 15 | | |
| Instrumental | 8 | | 11 | | | 11 | | 10 | | |
| Name related | 3 | | 2 | | | 3 | | 4 | | |
| Manipulable | 11 | | 11 | | | 14 | | 12 | | |
| *Face/arm/leg* | | | | | | | | | | |
| *face actions | 2 | | 2 | | | 1 | | 3 | | |
| *arm actions | 13 | | 12 | | | 14 | | 15 | | |
| *leg actions | 3 | | 0 | | | 2 | | 1 | | |
| *face&arm actions | 0 | | 1 | | | 1 | | 0 | | |
| *arm&leg actions | 0 | | 1 | | | 0 | | 0 | | |
| *"NA" actions | 2 | | 3 | | | 2 | | 1 | | |
| *Conjugation* | | | | | | | | | | |
| *first | 15 | | 15 | | | 13 | | 15 | | |
| *second | 3 | | 2 | | | 4 | | 3 | | |
| *third | 2 | | 3 | | | 3 | | 2 | | |

**Supplementary Table 9. Matching of treated and untreated verbs for psycholinguistic variables: PG**

| | Phase 1 | | | | | Phase 2 | | | | |
|---|---|---|---|---|---|---|---|---|---|---|
| | Untreated | | Treated | | T-test | Untreated | | Treated | | T-test |
| | *Mean* | *std* | *Mean* | *std* | *P-value* | *Mean* | *std* | *Mean* | *std* | *P-value* |
| Sentence agreement | 91.00% | 0.091 | 90.50% | 0.076 | 0.852 | 88.50% | 0.081 | 91.00% | 0.072 | 0.309 |
| Age of Acquisition | 2.135 | 0.470 | 2.036 | 0.473 | 0.511 | 1.942 | 0.429 | 2.002 | 0.633 | 0.729 |
| Imageability | 1.392 | 0.327 | 1.322 | 0.326 | 0.503 | 1.378 | 0.341 | 1.373 | 0.304 | 0.962 |
| Relative frequency | 27.897 | 42.606 | 27.432 | 53.424 | 0.976 | 45.614 | 45.994 | 38.532 | 73.549 | 0.717 |
| Length in phonemes | 8.100 | 1.373 | 8.200 | 1.240 | 0.810 | 7.800 | 1.281 | 8.050 | 1.468 | 0.570 |
| | *Count* | | *Count* | | | *Count* | | *Count* | | |
| Transitivity | 14 | | 14 | | | 15 | | 15 | | |
| Internal arguments | 14 | | 14 | | | 15 | | 15 | | |
| Instrumental | 10 | | 10 | | | 9 | | 6 | | |
| Name related | 5 | | 4 | | | 2 | | 1 | | |
| Manipulable | 13 | | 13 | | | 13 | | 11 | | |
| *Face/arm/leg* | | | | | | | | | | |
| *face actions | 2 | | 2 | | | 3 | | 1 | | |
| *arm actions | 14 | | 14 | | | 15 | | 12 | | |
| *leg actions | 1 | | 1 | | | 1 | | 0 | | |
| *face&arm actions | 0 | | 1 | | | 1 | | 2 | | |
| *arm&leg actions | 1 | | 0 | | | 0 | | 0 | | |
| *"NA" actions | 2 | | 1 | | | 0 | | 4 | | |
| *Conjugation* | | | | | | | | | | |
| *first | 17 | | 16 | | | 20 | | 18 | | |
| *second | 1 | | 2 | | | 0 | | 2 | | |
| *third | 2 | | 2 | | | 0 | | 0 | | |

**Supplementary Table 10. Matching of treated and untreated verbs for baseline accuracy and error types: LF**

| | Phase 1 (sum) | | Phase 2 (sum) | |
|---|---|---|---|---|
| | Sum | | Sum | |
| | Untreated | Treated | Untreated | Treated |
| Semantic paraphasia | 8 | 3 | 6 | 8 |
| Anomia (no response) | 31 | 36 | 32 | 29 |
| Phonemic paraphasia | 2 | 6 | 4 | 3 |
| Unrelated word | 1 | 3 | 1 | 4 |
| Word fragment | 0 | 0 | 1 | 2 |
| Neologism | 3 | 6 | 0 | 0 |
| Other | 5 | 3 | 3 | 4 |
| Baseline accuracy (max=60) | 2 | 6 | 11 | 9 |
| Comprehension errors (max=60) | 0 | 4 | 2 | 4 |

**Supplementary Table 11. Matching of treated and untreated verbs for baseline accuracy and error types: GC**

| | Phase 1 (sum) | | Phase 2 (sum) | |
|---|---|---|---|---|
| | Sum | | Sum | |
| | Untreated | Treated | Untreated | Treated |
| Semantic paraphasia | 24 | 26 | 15 | 12 |
| Anomia (no response) | 10 | 11 | 13 | 12 |
| Phonemic paraphasia | 1 | 2 | 1 | 0 |
| Unrelated word | 5 | 7 | 2 | 1 |
| Word fragment | 1 | 0 | 1 | 1 |
| Neologism | 0 | 2 | 0 | 0 |
| Other | 3 | 2 | 3 | 5 |
| Baseline accuracy (max=60) | 15 | 13 | 24 | 23 |
| Comprehension errors (max=60) | 5 | 5 | 1 | 1 |

**Supplementary Table 12. Matching of treated and untreated verbs for baseline accuracy and error types: GD**

| | Phase 1 (sum) | | Phase 2 (sum) | |
|---|---|---|---|---|
| | Sum | | Sum | |
| | Untreated | Treated | Untreated | Treated |
| Semantic paraphasia | 12 | 10 | 3 | 5 |
| Anomia (no response) | 19 | 17 | 16 | 19 |
| Phonemic paraphasia | 1 | 1 | 1 | 0 |
| Unrelated word | 1 | 2 | 0 | 0 |
| Word fragment | 1 | 0 | 2 | 1 |
| Neologism | 0 | 0 | 0 | 0 |
| Other | 1 | 1 | 2 | 4 |
| Baseline accuracy (max=60) | 17 | 19 | 23 | 24 |
| Comprehension errors (max=60) | 0 | 0 | 0 | 0 |

**Supplementary Table 13. Matching of treated and untreated verbs for baseline accuracy and error types: GP**

| | Phase 1 (sum) | | Phase 2 (sum) | |
|---|---|---|---|---|
| | Sum | | Sum | |
| | Untreated | Treated | Untreated | Treated |
| Semantic paraphasia | 9 | 18 | 22 | 23 |
| Anomia (no response) | 2 | 2 | 5 | 3 |
| Phonemic paraphasia | 2 | 1 | 4 | 3 |
| Unrelated word | 7 | 10 | 3 | 2 |
| Word fragment | 0 | 0 | 0 | 0 |
| Neologism | 1 | 0 | 0 | 0 |
| Other | 1 | 1 | 1 | 0 |
| Baseline accuracy (max=60) | 14 | 14 | 21 | 22 |
| Comprehension errors (max=60) | 0 | 0 | 0 | 0 |

**Supplementary Table 14. Matching of treated and untreated verbs for baseline accuracy and error types: EC**

| | Phase 1 (sum) | | Phase 2 (sum) | |
|---|---|---|---|---|
| | Sum | | Sum | |
| | Untreated | Treated | Untreated | Treated |
| Semantic paraphasia | 6 | 7 | 11 | 9 |
| Anomia (no response) | 20 | 17 | 17 | 18 |
| Phonemic paraphasia | 0 | 0 | 0 | 0 |
| Unrelated word | 3 | 3 | 3 | 4 |
| Word fragment | 2 | 1 | 1 | 0 |
| Neologism | 0 | 0 | 0 | 0 |
| Other | 8 | 9 | 11 | 7 |
| Baseline accuracy (max=60) | 11 | 11 | 17 | 17 |
| Comprehension errors (max=60) | 0 | 0 | 0 | 0 |

**Supplementary Table 15. Matching of treated and untreated verbs for baseline accuracy and error types: SP**

| | Phase 1 (sum) | | Phase 2 (sum) | |
|---|---|---|---|---|
| | Sum | | Sum | |
| | Untreated | Treated | Untreated | Treated |
| Semantic paraphasia | 7 | 4 | 9 | 9 |
| Anomia (no response) | 28 | 27 | 29 | 29 |
| Phonemic paraphasia | 1 | 3 | 1 | 1 |
| Unrelated word | 11 | 12 | 11 | 8 |
| Word fragment | 0 | 0 | 0 | 0 |
| Neologism | 5 | 6 | 2 | 4 |
| Other | 2 | 0 | 0 | 2 |
| Baseline accuracy (max=60) | 0 | 0 | 5 | 5 |
| Comprehension errors (max=60) | 6 | 7 | 8 | 7 |

**Supplementary Table 16. Matching of treated and untreated verbs for baseline accuracy and error types: RL**

| | Phase 1 (sum) | | Phase 2 (sum) | |
|---|---|---|---|---|
| | Sum | | Sum | |
| | Untreated | Treated | Untreated | Treated |
| Semantic paraphasia | 6 | 7 | 9 | 6 |
| Anomia (no response) | 2 | 2 | 1 | 0 |
| Phonemic paraphasia | 5 | 1 | 5 | 2 |
| Unrelated word | 0 | 3 | 0 | 1 |
| Word fragment | 5 | 3 | 4 | 9 |
| Neologism | 0 | 1 | 0 | 0 |
| Other | 11 | 15 | 0 | 0 |
| Baseline accuracy (max=60) | 1 | 0 | 39 | 39 |
| Comprehension errors (max=60) | 0 | 0 | 0 | 1 |

**Supplementary Table 17. Matching of treated and untreated verbs for baseline accuracy and error types: KC**

| | Phase 1 (sum) | | Phase 2 (sum) | |
|---|---|---|---|---|
| | Sum | | Sum | |
| | Untreated | Treated | Untreated | Treated |
| Semantic paraphasia | 8 | 9 | 6 | 9 |
| Anomia (no response) | 17 | 21 | 5 | 8 |
| Phonemic paraphasia | 2 | 3 | 1 | 3 |
| Unrelated word | 2 | 3 | 2 | 2 |
| Word fragment | 3 | 2 | 6 | 4 |
| Neologism | 0 | 0 | 0 | 1 |
| Other | 12 | 6 | 15 | 10 |
| Baseline accuracy (max=60) | 13 | 15 | 23 | 24 |
| Comprehension errors (max=60) | 2 | 0 | 3 | 3 |

**Supplementary Table 18. Matching of treated and untreated verbs for baseline accuracy and error types: PG**

| | Phase 1 (sum) | | Phase 2 (sum) | |
|---|---|---|---|---|
| | Sum | | Sum | |
| | Untreated | Treated | Untreated | Treated |
| Semantic paraphasia | 16 | 14 | 7 | 8 |
| Anomia (no response) | 2 | 1 | 0 | 1 |
| Phonemic paraphasia | 5 | 6 | 10 | 12 |
| Unrelated word | 3 | 4 | 2 | 2 |
| Word fragment | 4 | 9 | 3 | 6 |
| Neologism | 5 | 6 | 0 | 1 |
| Other | 15 | 16 | 13 | 7 |
| Baseline accuracy (max=60) | 9 | 9 | 35 | 34 |
| Comprehension errors (max=60) | 0 | 3 | 1 | 2 |
